# Estimation of pore structure and permeability in tight carbonate reservoir based on machine learning (ML) algorithm using SEM images of Jaisalmer sub-basin, India

**DOI:** 10.1038/s41598-024-51479-9

**Published:** 2024-01-09

**Authors:** Pydiraju Yalamanchi, Saurabh Datta Gupta

**Affiliations:** Department of Applied Geophysics, Indian Institute of Technology (Indian School of Mines), Dhanbad, 826004 India

**Keywords:** Geophysics, Fossil fuels

## Abstract

Analyzing the pore structure in carbonate reservoirs plays a crucial role in predicting fluid flow characteristics within these formations. The goal of the study was to use machine learning techniques for pore structure analysis and estimation of permeability in carbonate reservoirs. We implemented these algorithms by examining 2D scanning electron microscope (SEM) images of carbonate samples from the Jaisalmer sub-basin captured at various magnifications. In the initial stage of the analysis, various binarization algorithms were applied to determine carbonate sample porosity. Among these algorithms, the MaxEntropy algorithm gave a porosity value closely aligned with those obtained through petrography analysis. We employed the watershed algorithm to find the pore network parameters of carbonate samples at various magnifications. We observed that changes in magnification affected pore network parameters, resulting in a reduction in pore size distribution, throat radius, and grain size. Subsequently, we employed the numerical lattice Boltzmann method (LBM) to estimate the permeability of carbonate samples and compared to values derived from well logs. We employed machine learning (ML) algorithms, specifically Artificial Neural Network (ANN) and Support Vector Machine (SVM), to predict the permeability of carbonate samples. The input features for these models were the pore network parameters, while the LBM permeability values served as the output. We examine the prediction performance of these methods against the measured LBM permeability by conducting the error analysis and the coefficient of determination (${R}^{2}$) calculation. Our findings revealed that the ANN models outperformed the SVM models. Specifically, the ANN model displayed an impressive R^2^ value of 0.892, along with root mean square error (RMSE), mean squared error (MSE) and, mean absolute error (MAE) values of 1.927, 3.716 and 1.580, respectively. In contrast, the SVM model yielded an R^2^ value of 0.849, with RMSE, MSE and, MAE values of 2.324, 5.401 and, 2.166 respectively, when assessed on testing data of measured permeability. This study found that ANN is more dependable, robust, and precise than SVM in forecasting carbonate sample permeability.

## Introduction

Pore structure and permeability are crucial in the study of geoscience and petroleum engineering for oil & gas exploration. Pore structure and permeability play a crucial role in simulating fluid flow within the heterogeneous geometry of carbonate porous materials^1–5^. To investigate the single and multiphase fluid flow, pore network modeling and its characterization are crucial^6^. Permeability, which describes the flow of fluids through porous media, is one of the most important properties. Pore structure parameters, including porosity, tortuosity, connectivity, pore size, as well as pore shape and aspect ratio^7,8^ significantly influenced the permeability of porous media^7,8^. Several direct experimental approaches have developed to analyse the pore structure character and permeability of the porous medium. These approaches include mercury injection porosimetry (MIP), nuclear magnetic resonance (NMR), core analysis method developed by Gas Research Institute (GRI), and pulse decay^9–16^. Despite being frequently employed, these direct experimental measurements have significant limitations in current laboratory operating conditions, such as the involvement of significant time, cost-effectiveness, and a few cores samples^17^. These limitations motivate us to develop a new efficient algorithm for analyzing the pore structure characterization and permeability in a porous medium, which can produce reliable results within a short time and be cost effective.

In recent times, researchers widely accept imaging techniques such as X-ray micro-computed tomography and scanning electron microscopy (SEM) for imaging porous media. Incorporating numerical modelling approaches enhances the robustness of this technique to get proper pore structure, permeability, and fluid flow through porous media^1,18–20^. In between these methodologies, image processing plays a crucial role in characterizing the pore structure of porous media by extracting valuable quantitative information from microscopic images. This enables the precise evaluation of pore size, shape, distribution, and connectivity. This data is vital for understanding the physical properties of porous media, including porosity and permeability^5^. The most well-known direct simulation processes include the finite volume method (FVM)^21,22^, finite element methods (FEM)^23–26^, and the lattice Boltzmann method (LBM)^27–32^ are commonly employed for modelling porous media and simulating fluid transport process. Indirect strategies like Pore Network Modelling (PNM)^6,33–35^ and Bundle-of-Tubes^36^ offer effective means to simulate fluid flow behavior in porous media. These combined approaches contribute to a comprehensive understanding of porous media properties and fluid dynamics. The lattice Boltzmann method (LBM) has developed into the most capable and widely used numerical modelling approach for estimating the permeability and tortuosity of porous media^37^. It was started by Frisch et al.^38^ under the name of lattice–gas automata in 1986. The Lattice Boltzmann Method (LBM) is based on the mesoscopic physics of the Lattice Gas Cell Automata (LGCA)^39^. One can also derive the lattice Boltzmann method (LBM) directly from the discretized Boltzmann equation. The lattice Boltzmann method can predict an image-based throat permeability model with more accuracy. It uses thin sections of 2D SEM images or micro-computed tomography images are the input images^29^. The LBM simulations are valuable for studying the heterogeneity of carbonate reservoirs. It can accurately model fluid flow in complex, porous media with varying rock properties and enables the exploration of how small-scale variations in the carbonate rock matrix affect fluid flow, including the impact of different pore geometries and connectivity. This information is crucial for optimizing oil and gas recovery strategies, understanding reservoir performance, and predicting the behavior of fluids in heterogeneous carbonate reservoirs. These numerical approaches always require rigorous preparations for the discretization of porous media. To ensure the accuracy of the discretization result, high-quality digital images of porous media are always required. However, getting such images more costs are involved, and it is also time-consuming for geoscience and engineering applications. As a result, standard numerical simulation methods have significant limitations in accurately estimating the pore structure parameters of porous media. Another reason is that carbonate reservoirs are inherently diverse, making it challenging to predict the accurate permeability model using this numerical simulation and empirical methods^40^.

Because of the complexities involved in predicting permeability models for carbonate rocks, including the lack of high-quality images and heterogeneous carbonate samples, some researchers have introduced machine learning (ML) based algorithms. These algorithms provide accurate and reliable approaches to predicting the properties of porous media^41–43^. There are various ML algorithms focused on the reconstruction of porous media and estimating the pore network parameters, including porosity, permeability, tortuosity, throat radius, pore and grain size, etc. of the porous medium^44^. These algorithms particularly use X-ray micro-computed tomography and 2D SEM images, employing techniques such as Least Square Support Vector Machine (LSSVM), Fuzzy logic, K-means clustering, artificial neural network (ANN), genetic algorithm (GA) and conventional neural network (CNN)^45–48^.

Lu et al.^49^ developed a precise permeability prediction model tailored to distinct pore structure types of Cretaceous carbonate reservoir. The spectral coefficient method effectively discriminates between connected and unconnected pores. The alignment of fractal dimensions with pore structure characteristics serves as a validation of this pore structure classification. Cheng et al.^50^ employed a multiparameter equation derived via multiple regression analysis, comprehensively assessing the influence of pore-throat parameters at different scales on tight sandstone reservoir properties. It accurately predicts permeability and porosity, offering valuable insights for studying pore structure and permeability in tight sandstones. Adegbite et al.^51^ used the multiple linear regression analysis and Artificial Neural Network (ANN) to investigate the relationship between porosity, pore radius, throat radius, and permeability. They compared these findings to experimental results got at different levels of mercury saturation. The results revealed that the multiple linear regression technique exhibited the strongest correlation at 35% mercury saturation, whereas the ANN demonstrated a better correlation at 55% mercury saturation. These results highlight the superior performance of the ANN over multiple regression in permeability prediction. The utilization of the Fuzzy Logic method with wireline well log data from carbonate reservoirs in the Middle East yields the most accurate permeability model. These predicted models exhibit an exceptional agreement with core permeability. Rostami et al.^52^ employed various ML algorithms, including radial basis function neural network (RBF-ANN), least square support vector machine (LSSVM), multilayer perception neural network (MLP-ANN), genetic programming (GP) and committee machine intelligent system (CMIS), to achieve a precise estimation of permeability in a heterogenous carbonate reservoir. GP and CMIS models provided the most accurate predictions, showing the highest determination coefficient when the researchers compared the results with core permeability. Zhang et al.^53^ proposed the conventional neural network (CNN) approach based on the autoencoder (AE) effectively predicts permeability using low-resolution images of porous media where an autoencoder (AE) module trained with unlabelled data and CNN trained with a small amount of labeled data. The results show that this AE-CNN approach outperforms traditional CNN and lattice Boltzmann method (LBM) approaches, with an average R^2^ value of 0.896 and low mean-square errors, showing substantial improvements in prediction accuracy from low-resolution porous media images. Tran et al.^54^ used both ANN and multiple regression to investigate the indirect correlation between pore throat radius, permeability, and porosity of carbonate samples. Compared to multiple regression, ANN exhibited superior performance with a higher correlation factor in predicting permeability. The permeability prediction through numerical viscous flow simulation closely aligns with measured values when using 2D SEM images of porous media^4^. Gohari et al.^55^ successfully extracted pore-network parameters from 2D images of carbonate samples and accurately predicted the true permeability by using the ANN technique. Their finding showed promising results. Predicting 2D permeability from thin section images taken perpendicular to the plane and establishing statistical correlations with the computed 3D permeability of the host volumes resulted in accurate predictions^56^. In this study, we quantified the pore structure and permeability prediction by utilizing scanning electron microscopy (SEM) images taken at various magnifications (× 100, × 150, × 200, and × 300) of carbonate samples from the Kuldhar, Joyan, and Badabag members of Jaisalmer formation. The Jaisalmer limestone formation shows promise for hydrocarbon exploration. However, a lack of comprehensive data and geological complexities limits the comprehensive analysis of this algorithm in the Jaisalmer sub-basin, introducing uncertainties in the study of petrophysical properties of reservoir^57^. We quantified pore structure by estimating key pore network parameters including, porosity, pore size, throat radius, coordination number, and grain size. While the aspect ratio, especially valuable for distinguishing between elongated and spherical pores, prominently applies in scenarios where pore shape is a pivotal factor, the aforementioned parameters play a fundamental role in influencing fluid transport characteristics and permeability in porous media^58^.

The primary aim of this study is to predict the permeability of carbonate samples by using machine learning (ML) algorithms. Many studies have focused on predicting the permeability of 2D SEM images of carbonate samples using machine learning algorithms. Previous investigations have typically compared the predictive models against laboratory-based studies. In our study, we employed both numerical simulation and machine learning (ML) algorithms to estimate the permeability of carbonate samples permeability based on 2D SEM images at various magnifications. We examined the influence of different pore network parameters, such as pore network parameters, such as porosity, average pore radius, average throat radius, average grain size, and average coordination number, in different magnifications. Unlike previous studies that often-compared ML models to laboratory-based studies, we compared ML predictive models with numerical simulation methods. We employed Artificial Neural Network (ANN) and Support Vector Machine (SVM) techniques, using pore network parameters as inputs and lattice Boltzmann method (LBM) simulation results as targets to predict permeability. Our ML models yielded reliable permeability results for carbonate samples. Our study innovated by using the ANN technique, which generated precise and dependable permeability models for carbonate samples, regardless of the availability of core permeability values. The current research customized these methods to address geological and data challenges specific to the oil and gas industry, particularly in Indian carbonate reservoirs, enhancing their relevance and applicability in this context. It outlined a comprehensive workflow in the following section of the manuscript.

A notable innovation in our study is the utilization of the ANN technique, which provided accurate and reliable permeability models for carbonate samples, even where core permeability values were unavailable.

## Geology of the study area

The Jaisalmer basin, in the west part of the Aravalli ranges, stands the largest sub basin within the Rajasthan basin, covering an approximate area of 50,000 square kilometres. It’s divided into the northwest and the Barmer basin in the south by faults from the Bikaner-Nagaur basin. The basin exhibits pericratonic characteristics and comprises three depressions known as Shahgarh, Kishangarh and Miajlar depressions^59^. Three major unconformities are present in this basin. These unconformities delineate different stratigraphic sequences in this basin, such as Proterozoic–Early Cambrian, Paleozoic–Mesozoic, Tertiary, and Quaternary periods^60–62^. Many studies conducted in the Jaisalmer basin which shows the potentiality of hydrocarbon exploration in this basin^61,63,64^. The Jaisalmer basin comprises various ranges geological formations identified based on lithostratigraphy, spanning from the Eocene to Jurassic. These formations, namely Bandah, Goru, Habur, Pariwar, Baisakhi Jaisalmer, Lathi, Sanu and Khuiala are further classified into various members^65^. The underlying basement of this basin comprises rocks from the Precambrian era, which predominantly comprise igneous and metamorphic rocks.

**Figure 1:**
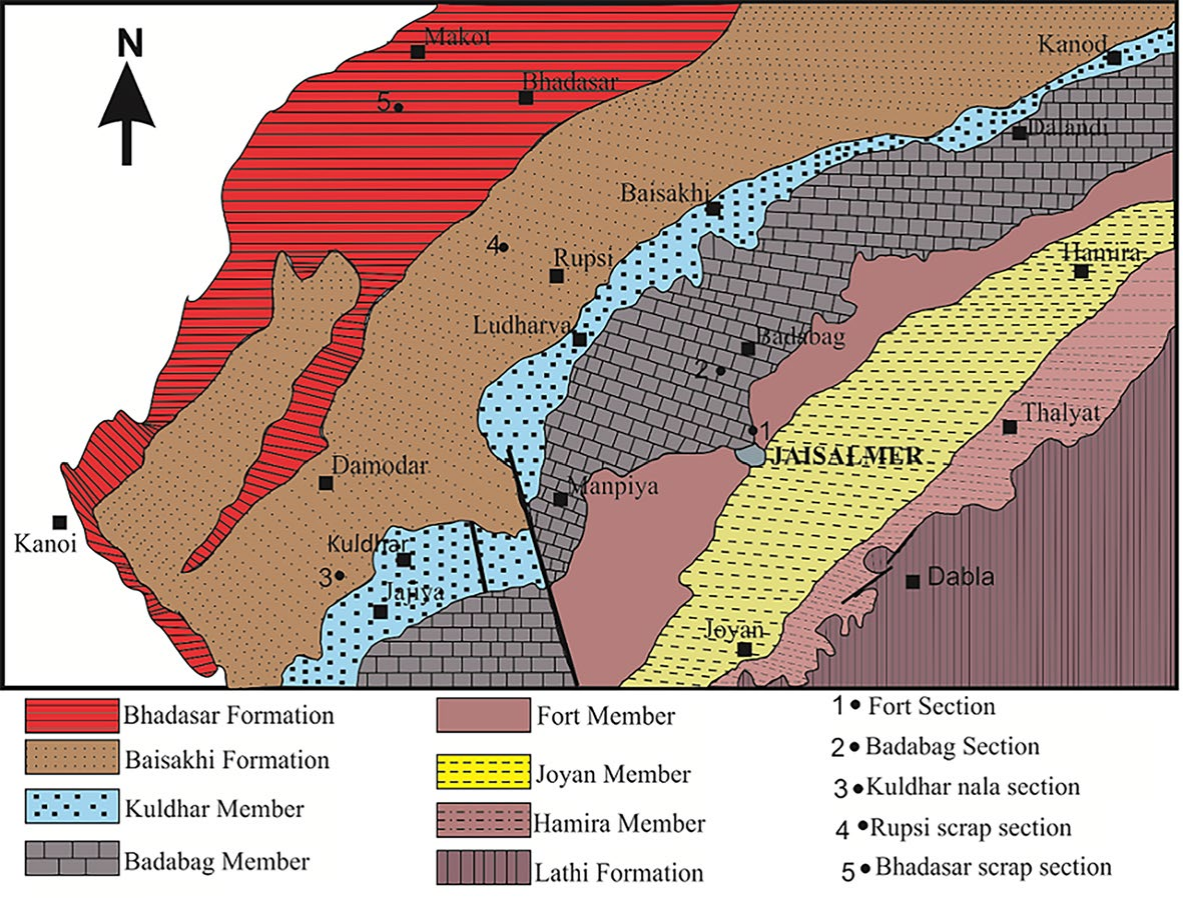
Geological map of the Jaisalmer sub-basin illustrating the various members of the Jaisalmer formation (modified after^66^).

Permian rocks are present in the Jaisalmer basin, which is a late Paleozoic–Mesozoic basin with an unconformable relationship with the Proterozoic basement. The basement of the basin primarily comprises the Malani suite, which is composed of Precambrian rocks, including metamorphic rocks. Jaisalmer basin consists thick sequence of sedimentary rocks such as clastic and carbonate formations. The Mesozoic rocks are well exposed in this basin, predominantly composed of limestone, shale, sandstone, and siltstone. The basement primarily comprises Pre-Cambrian rocks, notably the igneous and metamorphic rocks^62^. Several members, namely Hamira, Joyan, Fort, Badabag, Kuldhar, and Jajiya, categorize the Jaisalmer formation lithostratigraphically, which is well-known for its abundance of fossils. We can observe the outcrops of these members around the Jaisalmer city^66,67^. Figure 1 shows the different lithostratigraphic members of Jaisalmer formation. In this study, we used 2D SEM images of various carbonate samples of Jaisalmer formation to extract the pore network parameters and permeability prediction. The samples considered for analysis include those from the Hamira, Badabag and Kuldhar members. The lithostratigraphy of these members are illustrated in Table 1. Oolitic, cross-bedded limestone and sandstone are featured in the Jajiya formation. The Kuldhar formation comprises limestones, marls, and greenish shales. The Badabag formation comprises ferruginous sandstones and sandy limestones dating from the Middle to Late Bathonian period. The Joyan and Fort groups are primarily composed of sandstones and cross-bedded limestones. These formations contain corals that developed during the Early Bathonian and Bajocian periods. Last, the Hamira formation comprises limestones, calcareous sandstones, spanning from the Bajocian to the Early Jurassic^67–69^.

**Table 1:** Lithostratigraphy of the Jurassic strata of the Jaisalmer formation (modified after Sharma and Pandey^67^).

Formation	Member	Lithology	Age
Baisakhi	Lanela	Silty fine-grained sandstones	Tithonian–Oxfordian
	Rupsi	Bioturbated silty clay, fine grained sandstones	
	Basal	Carbonaceous, silty clay	
Jaisalmer	Jajiya	Oolitic, cross-bedded limestone, sandstone	Early to late Oxfordian
	Kuldhar	Limestones, marls, greenish shales	Callovian
	Badabag	Ferruginous sandstones, sandy limestones	Middle–Late Bathonian
	Fort	Fossiliferous limestones, medium grained sandstones	Early to Middle Bathonian
	Joyan	Shales, limestones with corals, sandstones	Early Bathonian–Bajocian
	Hamira	Limestones, calcareous sandstones	
Lathi	Thaiat	Sandstones with interbeds of shales, claystone	Bajocian–Early Jurassic
	Odania		

## Materials and methods

### Data preparation

This study is primarily based on carbonate samples of the Jaisalmer sub basin. We collected these samples from the Hamira, Badabag, and Kuldhar members of the Jaisalmer formation and conducted scanning electron microscope (SEM) tests on them. The SEM images were obtained at four different magnification levels: × 100, × 150, × 200, and × 300 using an acceleration voltage of 20 kV and a resolution of 647 × 486 pixels. 40 2D SEM carbonate samples from Jaisalmer formation were examined. To show the findings of this study, we present two representative carbonate samples, namely S-1 from the Kuldhar member and S-4 from the Badabag member. The selected samples are visible in different magnifications. Data from two wells, named A and B, were used to validate the simulation results. In this study, we used conventional well log data from these wells including gamma-ray (GR), resistivity (RT), density (RHOB), and neutron porosity (NPHI), along with estimated petrophysical logs such as porosity, permeability, and water saturation.

### Method

**Figure 2:**
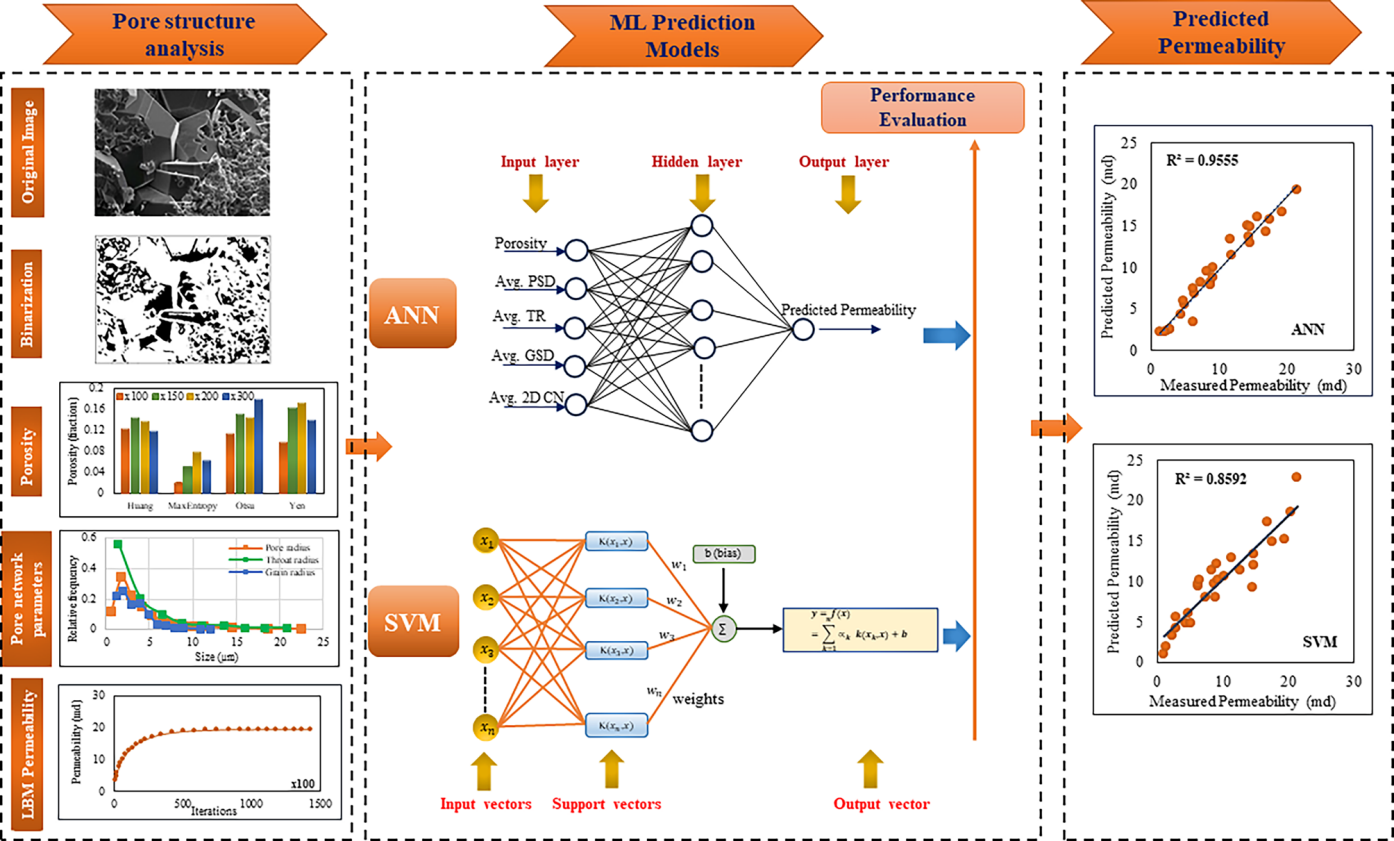
Schematic diagram of graphical work flow, which outlines the step-by-step workflow detailing the techniques employed in predicting the permeability of carbonate samples using machine learning (ML) models.

Our study focuses on predicting the permeability of carbonate samples using machine learning (ML) algorithms. Unlike previous studies that primarily compared ML models with laboratory experiments, we introduce a novel approach by comparing ML models with numerical simulation methods, particularly tailored for Indian carbonate reservoirs. Our method involves several key steps as illustrated in Fig. 2 which outlines the sequential steps and process involved. First, we quantitatively estimate the pore network parameters of carbonate samples at various magnifications. These parameters, including porosity, average pore radius, average throat radius, average grain size, and average coordination number, are determined using image processing techniques. Next, we estimate permeability through lattice Boltzmann method (LBM) simulations and validate these results against permeability values derived from well logs. Subsequently, we employed machine learning (ML) algorithms, specifically Artificial Neural Network (ANN) and Support Vector Machine (SVM) techniques, to predict the permeability of carbonate samples. The pore network parameters serve as inputs for these ML models, while the LBM simulation results serve as the target output. To ensure robust model performance, we employed the grid search algorithm and K-fold cross-validation while executing machine learning algorithms. We began by identifying the hyperparameters relevant to the ML models employed in our study. To systematically explore the hyperparameters we used a grid search approach. We defined a set of values for each hyperparameter that we wanted to optimize. During the modeling stage, we adopted the K-fold cross-validation technique to evaluate the performance of different hyperparameter combinations. We employed a K-value of 10 to ensure robustness in our results. We determined the optimal hyperparameters by evaluating the best performance achieved across all folds and selected them as the hyperparameters for our last model. Then we performed feature importance analysis for both ANN and SVM models. While these models do not inherently offer feature importance like decision tree algorithms, we employed distinct methods to access their feature relevance. For ANN, we used permutation importance and, for SVM, feature importance was determined by the magnitude of the feature coefficients. Finally, we identify the most effective ML model by evaluating the coefficient of determination and error matrices. Notably, our study innovatively uses the ANN technique, providing accurate and reliable permeability predictions.

#### Image processing

Image processing is necessary to analyze the characterization of the pore structure of 2D SEM images. It involves digital adjustments, conversion, and operations to extract valuable information, enhance visibility, and identify objects and patterns. Image processing techniques include image enhancement, noise reduction, and segmentation. Threshold segmentation plays a crucial role in image processing, as the quality of the image directly affects segmentation results. Hence, it is important to perform image enhancement and denoising prior to segmentation^5^.

**Figure 3:**
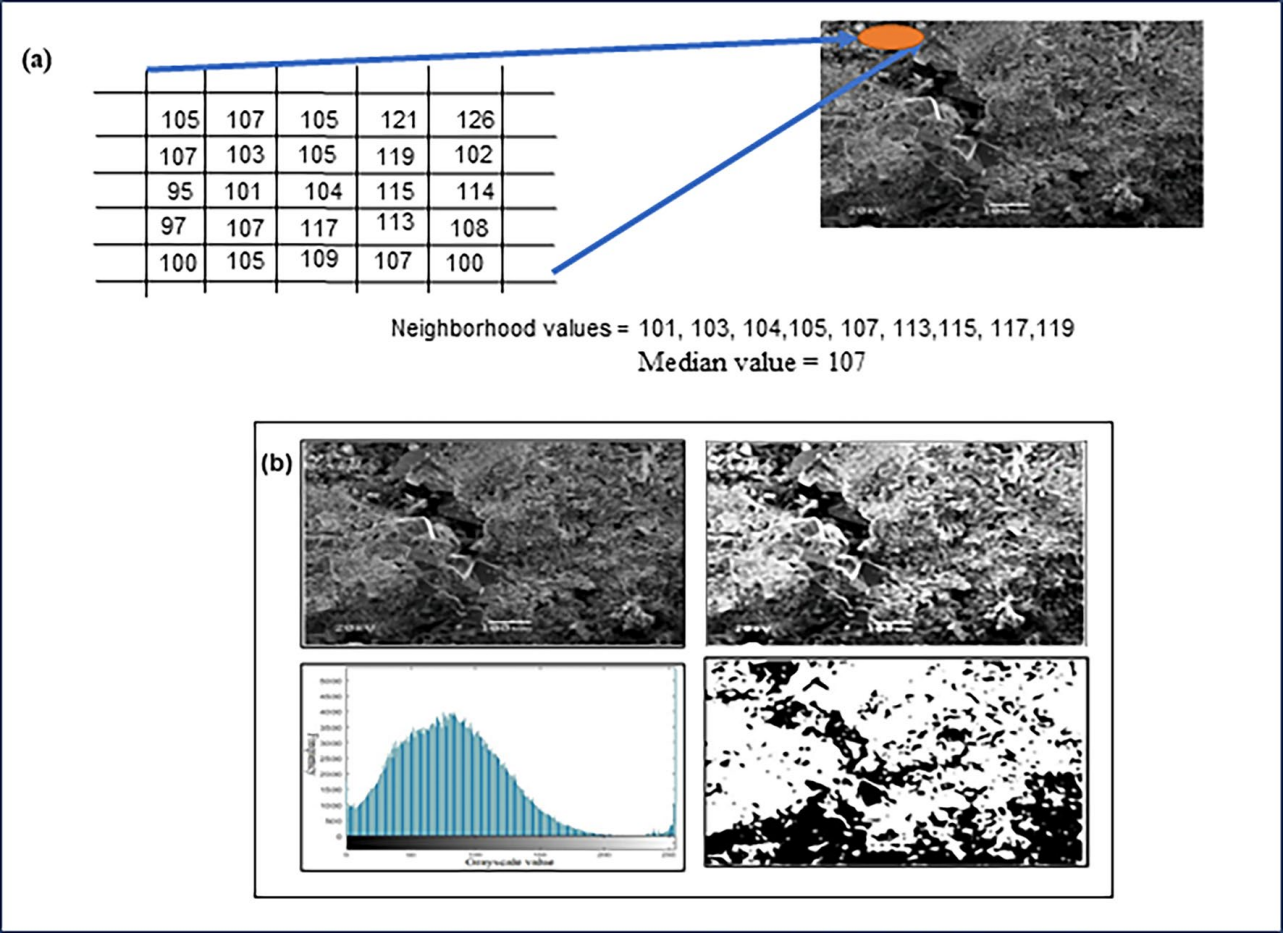
(**a**) Calculating the median value of pixel neighbourhood values for sample S-3. (**b**) The general workflow for image processing. The two figures above depict the original image and its enhancement, while the figure below illustrates the grayscale value distribution and binary image.

We accomplish image enhancement by adjusting the grayscale histogram of the image and expanding the dynamic range of grayscale values. The grayscale histogram represents the distribution of grayscale values, with the horizontal axis showing different gray levels and the vertical axis displaying the number of pixels at each gray level. Adjusting the histogram increases the dynamic range of grayscale values and enhances the contrast in the image. In an 8-bit grayscale image, which has 256 intensities, the histogram comprises 256 values representing the pixel distribution. Image denoising is another important process that aims to remove unwanted noise from an image. One effective method is the application of a median filter. The median filter is effective in reducing impulse noise and eliminating salt-and-pepper noise, while preserving the edges of an image. It works by sorting all pixel values within a neighbourhood and replacing the pixel value with the median (middle) value, rather than the average of the surrounding pixel values. Figure 3a illustrates the calculation of the median value of pixel neighbourhood for sample S-3. Image segmentation is a technique that partitions a digital image into distinct subgroups based on specific characteristics, simplifying image complexity, and facilitating further analysis by isolating the desired target. In this study, we focused on segmenting the pores in carbonate samples using a threshold-based segmentation algorithm^5^. The algorithm converts images into a binary format by representing object pixels with a single gray level and background pixels at different levels. Specifically, object pixels are assigned as “black”, while the background is represented as “white”. To perform threshold segmentation, it is essential to determine the threshold value using the following formula, which maps gray-level values to the binary set {0,1}. Equation (1) depicts the segmentation threshold value^5^.

1 $$\begin{aligned} S\left( {x,y} \right) = & 0,\; if\; f\left( {x,y} \right) < T\left( {x,y} \right) \\ = & 1, if\; f\left( {x,y} \right) \ge T\left( {x,y} \right) . \\ \end{aligned}$$

S (x, y) represents the value of the generated binary image, f (x, y) denotes the gray level of the original image, and T (x, y) indicates the threshold value of the segmented image at the coordinates (x, y). Figure 3b illustrates the image processing steps we performed on sample S-3.

#### Threshold-based segmentation algorithms

The threshold-based segmentation algorithms play a pivotal role in image segmentation, serving as a fundamental technique in the field. However, existing algorithms have limitations in effectively handling noisy grayscale images^5^. Researchers have devoted considerable attention to addressing these challenges over the past four decades. Two classifications of threshold segmentation are local thresholding and global thresholding, which involve dividing an image based on specific thresholds. Local thresholding divides the image into smaller sections and determines the threshold value for each section. Global thresholding involves determining a single threshold for the entire image. These algorithms employ various techniques, including histograms, clustering, entropy, and fuzzy logic-based methods to achieve their segmentation goals. In this study, we employed several global threshold algorithms, namely MaxEntropy^70^, Otsu^71^, Huang^72^, and Yen^73^ for image segmentation. This allowed for a comprehensive evaluation of their effectiveness and performance in our specific context.

The MaxEntropy approach is rooted in the maximization of measured information between the object and background within an image, with entropy serving as the metric for information measurement. However, the computational complexity associated with the MaxEntropy algorithm prompted the introduction of the maximum correlation criterion (MCC) by Yen^73^. This criterion, known as the Yen algorithm, facilitates the calculation of an optimal image threshold. The Huang algorithm proposed by Huang and Wang^72^, aims to reduce the fuzziness measures of an input image in order to determine the optimal threshold value. The concept of fuzziness in this context typically refers to the degree of fuzziness exhibited by a fuzzy set. Fuzziness is quantified using entropy based on the Shannon function from information theory. The Otsu algorithm uses the maximum inter-class variance between background and target images. By maximizing the separability of resulting classes at different gray levels, the Otsu algorithm enables effective threshold determination^71^.

#### Determination of pore network parameters

**Figure 4:**
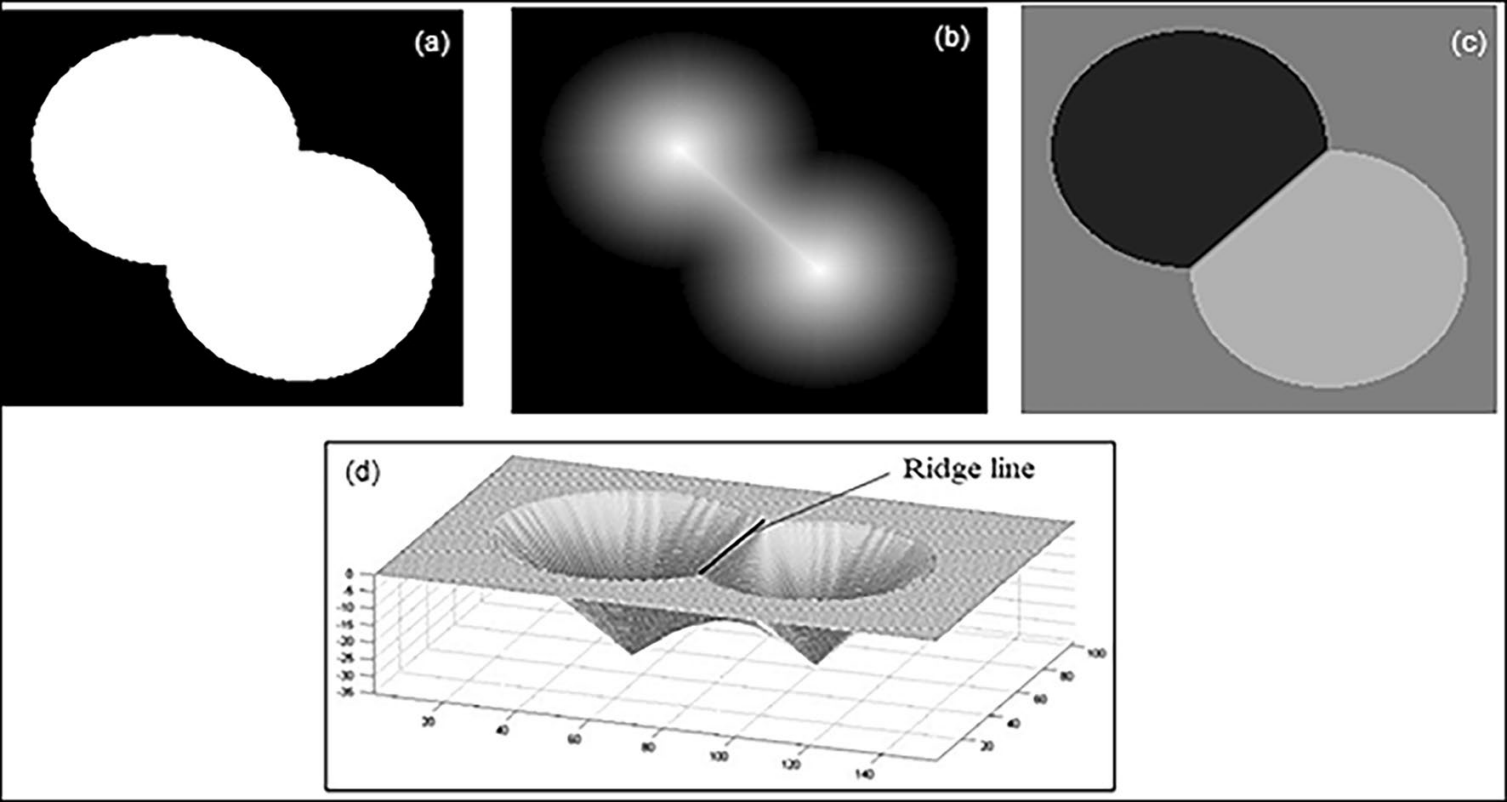
Schematic diagram of image segmentation with the watershed algorithm (*Source* Rabbani et al.^80^). (**a**) Original binary image with overlapping objects. (**b**) Grayscale distance transform of binary image, (**c**) watershed transform with segmented pores. (**d**) Catchment basins with watershed ridgeline. In the initial stage computes gradients within the image to identify potential markers, then markers are generated based on the gradient information to define regions of interest, in the next stage the watershed transform is applied to the marker image to segment the image into distinct regions using marker-based information. In final stage displays the resulting segmentation, highlighting different regions of the image based on watershed transformation.

To comprehend the flow characteristics of porous media, including important factors such as permeability, relative permeability, and fluid flow phenomena, it is crucial to understand the pore structure parameters that occur when the porous material is subjected to pressure differentials. Disparities in pore structure, such as spatial distribution, connectivity types, and pore and throat shapes and sizes, can lead to distinct fluid flow behaviours even when two materials have the same porosity. Therefore, accurate characterization of pore structure is essential, particularly in domains like petroleum and reservoir engineering, where pore network configuration and fluid flow dynamics significantly impact hydrocarbon storage capacity. In this study, we estimated various pore network parameters at different magnifications and thoroughly examined their influence on permeability prediction. In order to determine the 2D pore network parameters of carbonate samples, we employed the watershed algorithm^74^, initially introduced by Baldwin et al.^75^. Implementing this method used MATLAB’s educational image processing tool and the open-source ImageJ software^76–79^. In the watershed algorithm initially binarize the image as solids and voids, then obtain a distance map by calculating the minimum distance from the void to the nearest solid. Using this distance map, we segmented pores based on the concept of water flooding. In the calculated distance map, we considered the brightest colour indicating the lowest points. When simulating water flooding, water accumulates first at the lowest points within each water pool and gradually rises. When water from the different pools meets, the meeting point becomes the boundary between these pools. In this analysis, as all regions are filled with water, a series of meeting points forms a boundary line between the pools. This line is considered as the meeting line between the two pores, and as it coincides with the construction between two pores, it serves as the throat. This distinction allows us to analyze the pore-throat network effectively using 2D images^80^. Figure 4 illustrates the various stages of the watershed segmentation process.

The pore size distribution (PSD) and throat radius are influential parameters in the flow dynamics of porous media. To determine pore-throat sizes in 2D or 3D digital images, a promising approach uses the city-block distance function and watershed segmentation^80,81^. The method applies the morphological majority transform function to a binary image, reducing the roughness and noise for accurate PSD determination. The computed city-block distance produces sharp contour lines that delineate pore boundaries, with the brightest lines representing the lowest points. Watershed segmentation then connects marked points to generate ridgelines, enabling identification of the pore area above a specific radius. The contour line where two pores meet shows the throat, representing the constricted region between them.

In a pore network model, the 2D coordination number represents the average number of throats connected to each pore or the number of pore bodies associated with a single pore^3^. This parameter plays a crucial role in characterizing the pore network and has a substantial influence on the hydraulic conductivity of porous rocks. The average 2D coordination number is determined by calculating the mean number of throats connected to a specific pore. We employ the watershed segmentation algorithm to identify the throats associated with each pore, enabling the computation of this important network property.

To determining the grain size distribution (GSD) from digital images involves the detection of overlapping grains and individual size calculation. In this study, we used a watershed algorithm applied to 2D binary images to determine the GSD. The process involved several steps. The first step was to calculate the Euclidean distance between each pixel and its nearest pore pixels. The H-minima transform prevents over-segmentation by the watershed algorithm. This transform helped identify the ridge line between each pair of local minima in the distance image. Finally, GSD was determined along the principal axes of the images. For the 2D images, the GSD was measured along the vertical axes^82^.

### Permeability estimation based on LBM simulation

The goal of this work is to develop a machine learning (ML) based permeability prediction models for 2D SEM images of carbonate samples. However, when core permeability data is unavailable for training the ML models, traditional numerical methods are still necessary. lattice Boltzmann method (LBM) is the most reliable tool for permeability calculation among other traditional numerical methods^83^. It offers an alternative approach for simulating incompressible fluid flow. In our study, we assume a throat with a random cross-section and a consistent shape along its length to represent single-phase steady-state flow in porous media. Therefore, the permeability of the throat can be determined without simulating the entire length. Equation (2) expresses the lattice Boltzmann equation, which is connected to the Boltzmann equation^84^.

2 $${f}_{i }\left(x+ {c}_{i}\Delta t, t+\Delta t\right)- {f}_{i}\left(x, t\right) = {\delta }_{i}.$$

Here, the particle distribution function is ${f}_{i}$, the particle velocity is ${c}_{i}$ in the ith direction, and the collision operator is ${\delta }_{i}$.

In this study, it was expected that the particle distribution would interact with fluid bounce-back boundary conditions. For each time step ($t+\Delta t$) and spatial location $x$, the evolution of the discretized particle distribution function ${f}_{i}$ in accordance with the particle distribution velocities ${c}_{i}$ was determined using the Bhatnagar, Gross, and Krook (BGK) collision model^85^. Equation (3) illustrates this model.

3 $${f}_{i }\left(x+ {c}_{i}\Delta t, t+\Delta t\right)- {f}_{i}\left(x, t\right)= -\frac{1}{\tau }\left[{f}_{i}\left(x, t\right)- {f}_{i}^{eq}\left(x, t\right)\right].$$

In this context, $\tau$ represents the dimensionless relaxation time, $\Delta t$ denotes the time step, and the right-hand side term corresponds to the SRT-Bhatnagar, Gross, and Krook (BGK) collision model^85^. The equilibrium distribution function is ${f}_{i}^{eq}\left(x, t\right).$ Equation (4) shows the formulation of the distribution function at equilibrium as a function of velocity (ϑ) in all directions.

4 $${f}_{i}^{eq}\left(x, t\right)= {\omega }_{i}\rho + \rho {s}_{i}\left(\vartheta \left(x, t\right)\right).$$

The macroscopic quantities of density $\rho$ and velocity $\rho (\vartheta )$ are calculated using the particle distribution function ${f}_{i}$ as shown in Eqs. (5) and (6).

5 $$\rho = {\sum }_{i}{f}_{i},$$

6 $$\rho \left(\vartheta \right)= {\sum }_{i}{c}_{i}{f}_{i},$$

and ${s}_{i}(\vartheta )$ is defined in Eq. (7).

7 $${s}_{i}\left(\vartheta \right)= {\omega }_{i}\left[3\frac{{c}_{i}.\vartheta }{c}+ \frac{9}{2} \frac{{\left({c}_{i}.\vartheta \right)}^{2}}{{c}^{2}}- \frac{3}{2} \frac{\vartheta .\vartheta }{{c}^{2}}\right],$$

where $\vartheta$ represents the velocity vector, ${\omega }_{i}$ denotes the weight associated with velocity ${c}_{i,}$ and $c$ represents the sound speed.

**Figure 5:**
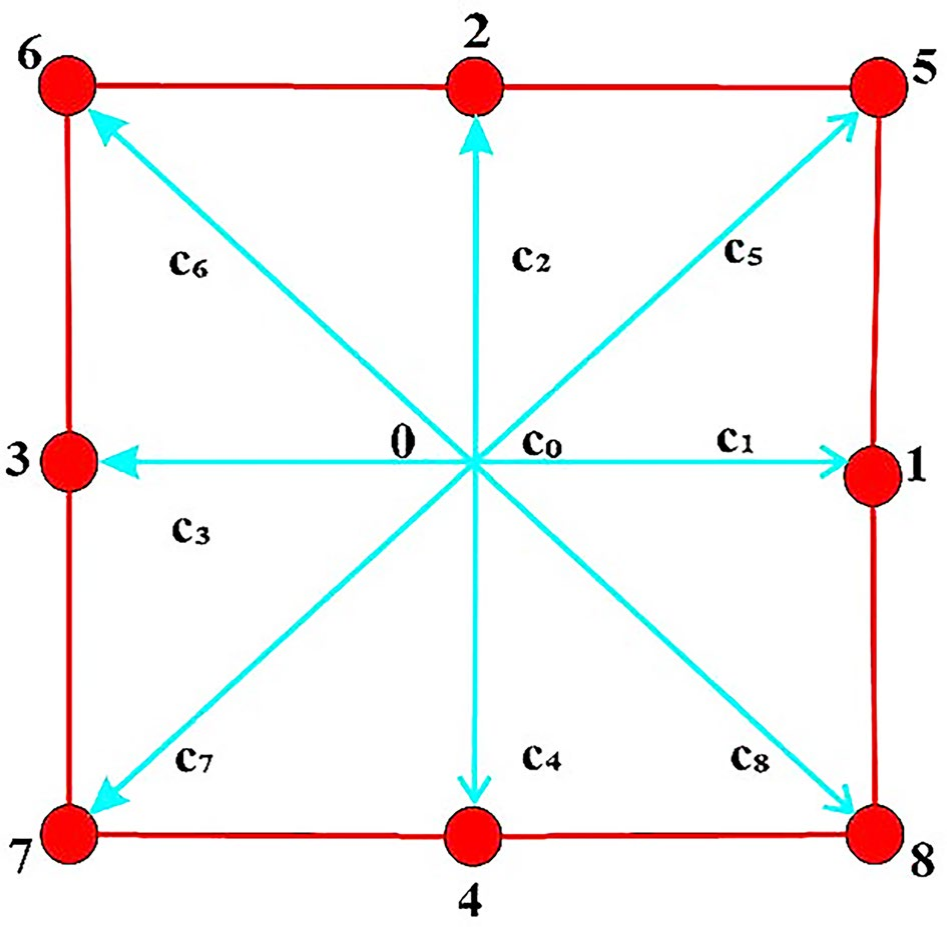
Schematic diagram of the D2Q9 configuration, which is utilized for the space discretization in the Lattice Boltzmann Method (LBM).

In this study, we used open-source LBM MATLAB programming to estimate the throat permeability of the porous medium, following the method proposed by Haslam et al.^86^. The study employs a D2Q9 model comprising nine discrete velocity vectors, representing potential fluid flow paths, as shown in Fig. 5. This approach applies to 2D digital images got from scanning electron microscopy (SEM) to determine the throat permeability.

The D2Q9 model has the nine velocity vectors ${c}_{i}$

$${c}_{i}= \left\{\begin{array}{l}\left(0, 0\right)\quad i=0 \\ \left(1, 0\right), \left(0, 1\right), \left(-1, 0\right), \left(0,-1\right) \quad i=1, 2, 3, 4\\ \left(1, 1\right), \left(-1, 1\right), \left(-1, -1\right), \left(1,-1\right) \quad i=5, 6, 7, 8\end{array}.\right.$$

For stationary, nearest and next-nearest vectors, the weight coefficients are ${\omega }_{0}=\frac{4}{9}, {\omega }_{1}= ----={\omega }_{4}=\frac{1}{9}, {\omega }_{5}= ----= {\omega }_{8}=\frac{1}{36}$ respectively.

In this flow simulation, the lattice BGK model applies to solve the steady-state planar Poiseuille equation, which describes the pressure drop in an incompressible fluid flowing through a cylindrical pipe with a constant cross-section under laminar flow conditions. Flow simulation is started by imposing a uniform distribution of vectors at the inlet channels of the geometry. The LBM simulation considers periodic boundary conditions in the flow directions, with the velocity distribution in the outlet channels, set equal to that in the inlet channels. We calculate the LBM permeability of throats in the porous medium as $\left(k= \frac{{r}^{2}}{8}\right)$ for laminar flow, where r represents the throat radius. This formula is independent of tube length and is applicable due to the reasonable assumption of periodic boundary conditions for the considered geometry. The simulation continues until distribution vectors at each channel reach equilibrium, equilibrium showing permeability convergence^87^.

At each iteration, we computed the permeability of throat tubes of the porous medium using Darcy’s law, rearranged as shown in Eq. (8).

8 $$k= -\frac{\mu U}{\left(\frac{dp}{dx}\right)},$$

where U is the mean velocity vector in the entire flow domain (toward pressure drop), $k$ is the throat permeability, $\frac{dp}{dx}$ is the pressure gradient, and $\mu$ is the fluid viscosity, Eq. (9) illustrates this calculation.

9 $$\mu = \frac{\frac{1}{\omega }-0.5}{3}.$$

Here $\omega$ is a relaxation frequency that is used in the LBM simulation, set to 1 to ensure convergence and minimize errors. In this study, throughout the simulation, the permeability data are recorded as numerical values representing the permeability of the throat tube after multiple iterations. This approach enables the computation of permeability and offers insights into the flow behavior within the porous medium^88^.

### Permeability determination from well log data

Several empirical methods have been proposed to determine permeability from well logs, which rely on establishing correlations between porosity, permeability, and irreducible water saturation^89–91^. In this study, we employed the Timur^90^ relationship to estimate permeability. In this study, we used data from two wells that were equipped with conventional logs, each providing measurements with a resolution of 0.125 m from top to bottom. The logs included Gamma Ray (GR), Resistivity (RT), Density RHOB), and Neutron Porosity (NPHI) logs. These logs were essential in our analysis. Specifically, we leveraged the porosity logs (Density and neutron porosity) to compute the porosity values. These porosity values played a crucial role in determining the irreducible water saturation. To achieve this, we applied a formula introduced by Buckles^92^ and later changed by Holmes et al.^93^. This changed formula suggested that the product of porosity and irreducible water saturation in a formation remains constant. The following equations were used to illustrate the calculation of permeability based on the Timur^90^ relationship. Equation (10) illustrates the irreducible water saturation calculation.

10 $${\mathrm{\varnothing }}^{Q}\times {S}_{wirr}=C,$$

where $\mathrm{\varnothing }$ represents the porosity, expressed as fractions. $Q$ is the porosity exponent, a dimensionless value that can range from 0.8 to 1.3, according to Holmes. In many reservoirs $Q=1$, which corresponds to the original Buckles formula. ${S}_{wirr}$ denotes the irreducible water saturation, also expressed as a fraction. $C$ represents the Buckles constant, a dimensionless value (sandstones = 0.02–0.10, Inter-granular carbonates = 0.01–0.06, Vuggy carbonates = 0.005–0.06). The permeability was estimated using Eq. (11).

11 $$K=0.136\frac{{\mathrm{\varnothing }}^{4.4}}{{S}_{wirr}^{2}} \left({\text{Timur}}, 1968\right),$$

where $K$ represents the permeability of formation, expressed as milli Darcy (md), S_wirr_ represents irreducible water saturation and ∅ represents the porosity.

### Machine learning algorithms

#### Artificial Neural Network (ANN)

**Figure 6:**
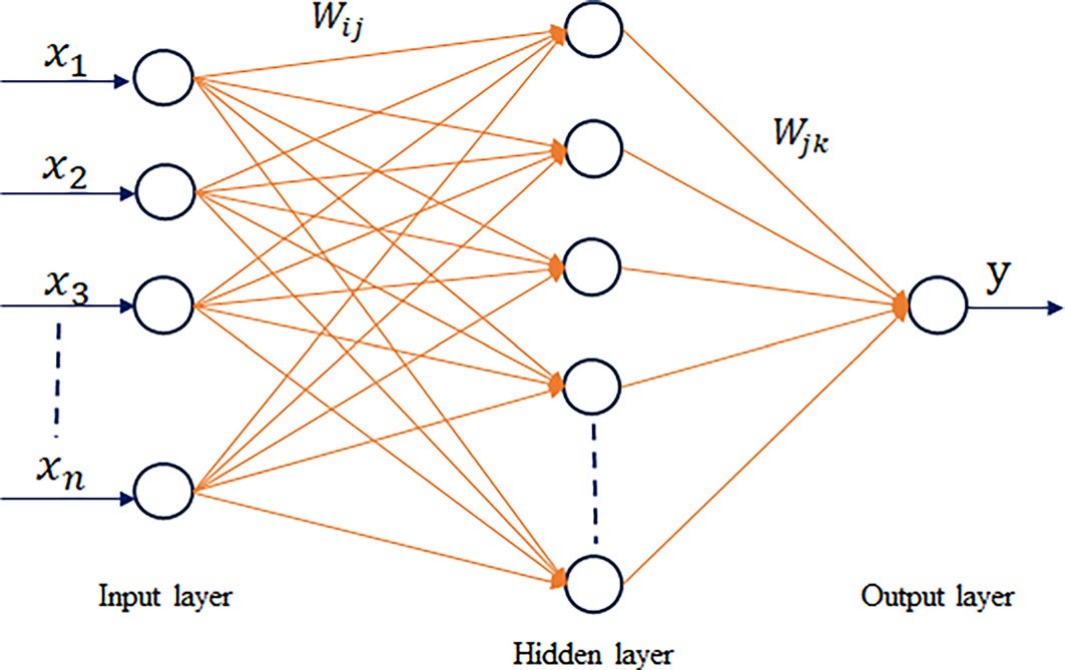
Schematic representation of the Artificial Neural Network (ANN). In this diagram, ${x}_{1}$, ${x}_{2}$…${x}_{n}$ represent the input parameters, while ${W}_{ij}$ and ${W}_{jk}$ denote weighted parameters applied to the input, connecting the input layer to hidden layer and the hidden layer to the output layer. The resulting output parameter is denoted as y.

Artificial Neural Networks (ANNs) are a class of machine learning models that draw inspiration from the structure and functioning of the human brain. They aim to replicate the processing and interpretation of information observed in biological neurons. Various fields have successfully applied ANNs, including image and speech recognition, natural language processing, and decision-making systems. ANNs gained significant popularity due to their ability to draw inspiration from the structure and functioning of the human brain^94^. An ANN comprises interconnected artificial neurons, referred to as nodes or units. These nodes are organized into layers, typically comprising an input layer, one or more hidden layers, and an output layer (see Fig. 6). Each node receives input signals, performs a computation, and generates an output signal that is then transmitted to nodes in the subsequent layer. The connections between nodes are represented by weights, which determine the significance of input signals in the overall computation. During the training phase, ANN learns to adjust these weights by iteratively processing the training data and comparing the predicted outputs with the desired outputs. This process, known as backpropagation, uses optimization algorithms to minimize the discrepancy between predicted and actual outputs, improving the network’s performance^95^. Determining the number of hidden layers is a key challenge in the application of neural network methods, and various approaches have been proposed to address this issue. Ham and Kostanic^96^ suggested employing a trial-and-error method to determine the optimal number of hidden layers. Several studies have shown that one or two hidden layers can effectively handle complex problems^97,98^.

In this study, we used the average pore network parameters as input variables to train an artificial neural network (ANN) model, which comprises a single hidden layer. This configuration enables the prediction of permeability based on the provided parameters. The input parameters comprise porosity, average pore size distribution, average throat size, average grain size, and average 2D coordination number. A stepwise approach was used to determine the optimal number of neurons in the hidden layer. Using a stepwise approach, we determined the optimal number of neurons in the hidden layer by measuring the error at each step while varying the number of neurons from 1 to 20. The ANN is trained using the Levenberg–Marquardt method with the MATLAB neural fitting tool. The trainer conducts the training process iteratively to achieve the best possible results.

#### Support vector machine (SVM)

Support vector machine (SVM) algorithm, introduced by Vapnik^99^ in 1995, is a widely used machine learning technique for both classification and regression tasks. The name given to the application of SVM for regression is Support Vector Regression (SVR). SVR follows the same principle as support vector classification, aiming to find a mapping function that relates the input features to the target variable. SVM has been successfully applied in various prediction problems across different domains^100^. However, unlike traditional regression models, SVR can capture nonlinear relationships between the features and the target variable by utilizing a Kernel function. The kernel function plays a crucial role in SVR by transforming the input features into a higher-dimensional space. By transforming the problem from nonlinear to linear, the optimal solution can be found^101^. The choice of kernel depends on the dataset and the complexity of the underlying relationship.

**Figure 7:**
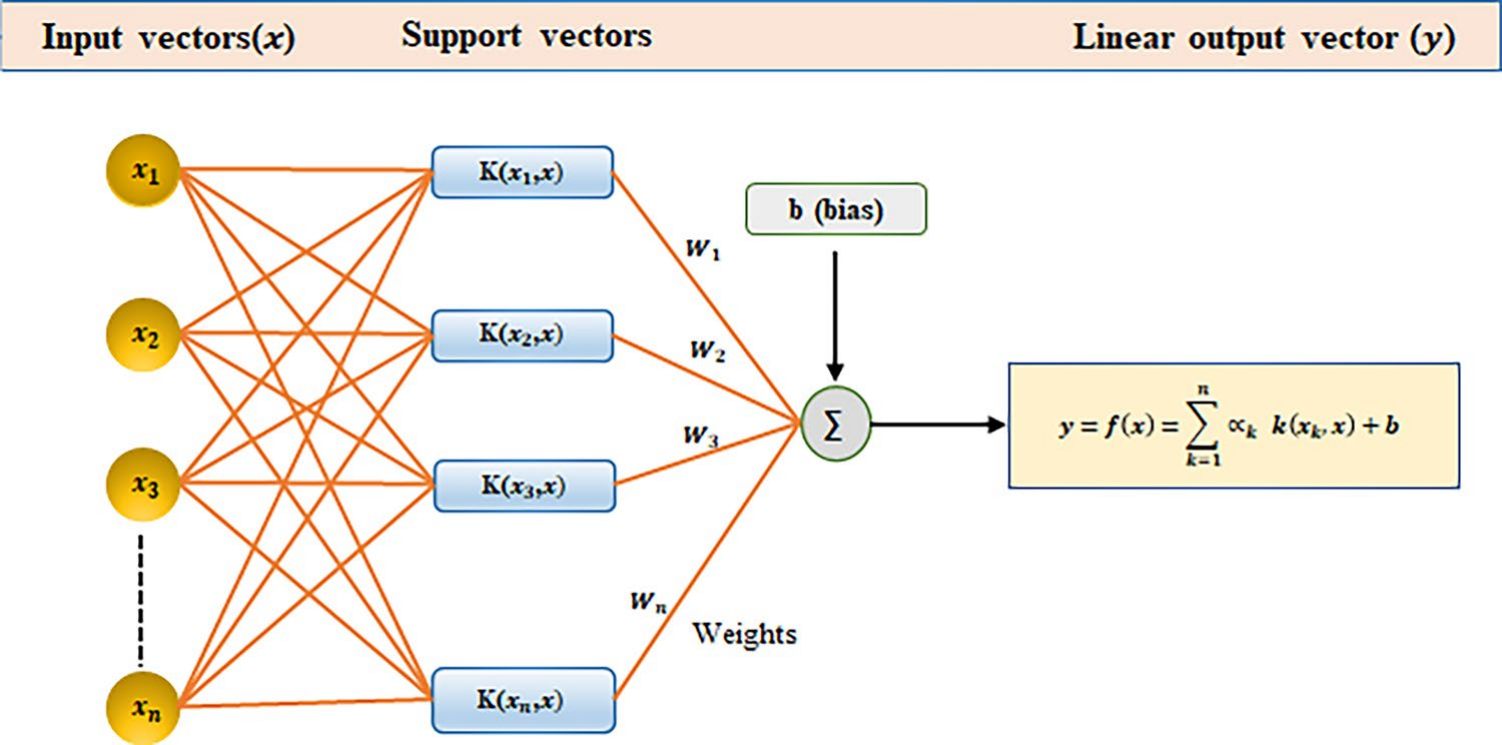
Schematic of the Support Vector Machine (SVM) method. The coefficients of w and b are the adjustable model parameters.

Let’s consider a training dataset denoted as $T=\left\{\left({x}_{1},{y}_{1}\right)\dots \left({x}_{n},{y}_{n}\right)\right\}$, where ${x}_{i}$ is the input vector and ${y}_{i}$ is the output vector. The SVM regression problem can be mathematically represented as shown in Eq. (12)^101^.

12 $$\begin{aligned} f\left( x \right) = & \mathop \sum \limits_{i = 1}^{n} \left( { \propto_{i} - \propto_{i}^{*} } \right) \cdot \phi_{{(x_{i} )}} \cdot \phi_{\left( x \right)} + b \\ = & \mathop \sum \limits_{i = 1}^{n} \left( { \propto_{i} - \propto_{i}^{*} } \right) \cdot k(x_{i} ,x) + b, \\ \end{aligned}$$

where ${\propto }_{i}$ and ${\propto }_{i}^{*}$ are the Lagrange multipliers associated with the corresponding input parameters, $k{(x}_{i},x)$ represents the kernel function, ${\varnothing }_{{(x}_{i})}$ and ${\varnothing }_{\left(x\right)}$ represent the transformed feature vectors in the higher dimensional space, and $b$ is the bias term. A typical architecture of SVM regression is depicted in the Fig. 7.

In this study, SVM regression analysis was conducted using various kernels, including linear, cubic, quadratic, fine Gaussian, medium Gaussian, and coarse Gaussian. The root mean square error (RMSE) and mean absolute error (MAE) values were determined for each SVM model with different kernels, and the kernel yielding the lowest RMSE and MAE values were selected as the most appropriate choice. We obtained RMSE and MAE values for each SVM model with different kernels and presented them in Table 2. After careful evaluation, we identified the linear kernel function for permeability prediction in this study. To optimize the SVM model for determining permeability, we employed a grid search approach to determine the most effective tuning parameters. Specifically, we selected the regularization parameter (c) of 10 and kernel-specific parameter gamma (γ) of 0.1 while utilizing the linear kernel function.

**Table 2:** Comparison of RMSE and MAE error values of different kernel functions of SVM method.

Kernal function	RMSE	MAE
Linear	2.86	2.151
Quadratic	3.34	3.103
Cubic	4.22	3.061
Fine Gaussian	5.60	4.526
Medium Gaussian	3.64	3.008
Coarse Gaussian	3.57	2.816

The Kernal function, which was used in this investigation, had the lowest RMSE and MAE value.

### Relative influences of the input variables on LBM permeability

Spearman’s rank correlation was used to analyse pore network parameter sensitivity to permeability estimation. Using Eq. (13)^102^, we calculated the Spearman’s correlation coefficient (ρ) within the range of − 1 to + 1 to determine the strength and direction of monotonic relationships between input and output variables in our model.

13 $$\rho = \frac{\sum_{i=1}^{n}\left({T}_{i}-\overline{T }\right)({Q}_{i}-\overline{Q })}{\sqrt{\sum_{i=1}^{n}{({T}_{i}-\overline{T })}^{2} \sum_{i=1}^{n}{({Q}_{i}-\overline{Q })}^{2}}}.$$

**Figure 8:**
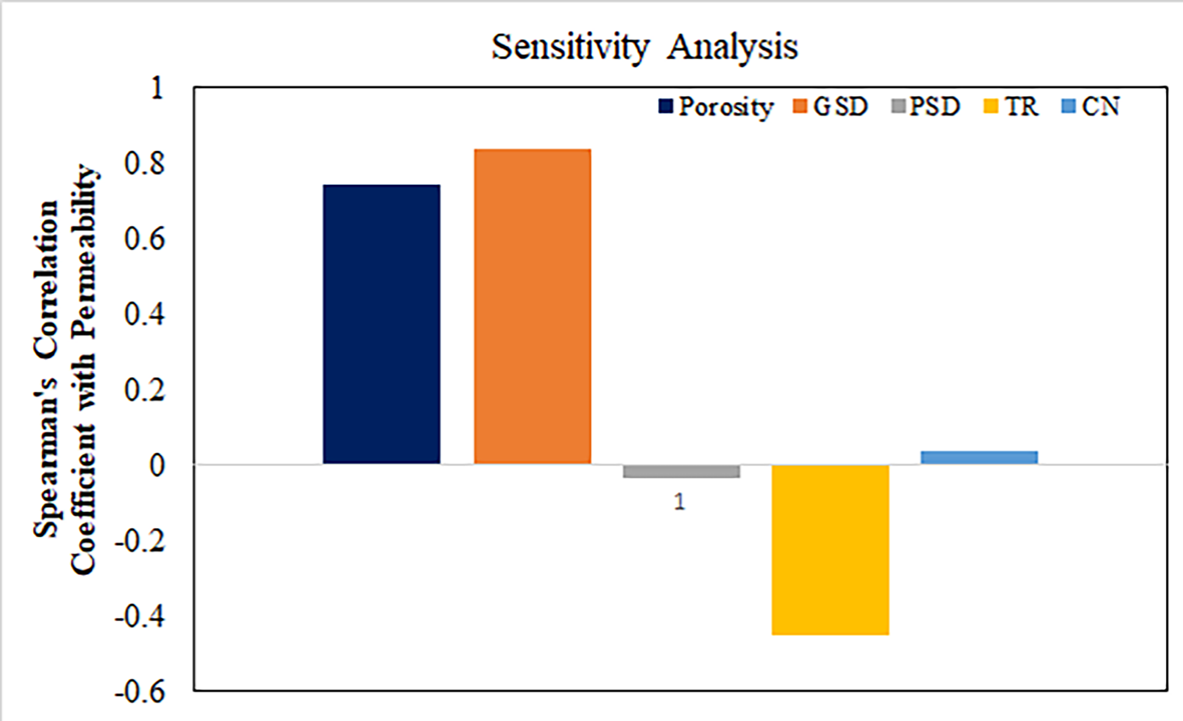
LBM permeability relationships with input variables assessed based on Spearman’s correlation coefficient values calculated for all datapoints of the input variables.

Figure 8 illustrates the impact of input variables on the output variable. Notably, grain size distribution (GSD) exhibited the most influence on LBM permeability, signifying its paramount importance. Conversely, pore size distribution (PSD) demonstrated the least influence, porosity and throat radius (TR) also displayed a significant impact on permeability, underscoring their relevance in the analysis.

### Model evaluation metrics

Model evaluation metrics in machine learning are used to assess the performance and quality of the trained model. This metrics provide quantitative measurements that help in understanding how well the model is performing and how accurately it is estimating predictions to meet the desired objectives. In this study, the most common evaluation metrics are used, include the coefficient of determination (${R}^{2}$), Root Mean Square Error (RMSE), Mean Square Error (MSE), and Mean Absolute Error (MAE). Equations (14)–(17) are used to calculate using these parameters.

14 $${R}^{2}=1- \frac{\sum_{i=1}^{n}{\left({X}_{i}-{Y}_{i}\right)}^{2}}{\sum_{i=1}^{n}{\left(\overline{Y }-{Y}_{i}\right)}^{2}},$$

15 $$\mathrm{MSE }= \frac{1}{n}\sum_{i=1}^{n}{({X}_{i}-{Y}_{i})}^{2},$$

16 $$\mathrm{RMSE }=\sqrt{\frac{1}{n}\sum_{i=1}^{n}{({X}_{i}-{Y}_{i})}^{2}},$$

17 $$\mathrm{MAE }= \frac{1}{n}\sum_{i=1}^{n}\left|{X}_{i}-{Y}_{i}\right|,$$

where ${X}_{i}$ and ${Y}_{i}$ are the predicted and measured values respectively, $\overline{Y }$ is represents the mean of the actual values and $n$ is the number of samples. According to previous studies, high coefficient of determination and minimal error values are showing high efficiency models.

## Results and discussion

### Segmentation of carbonate samples

**Figure 9:**
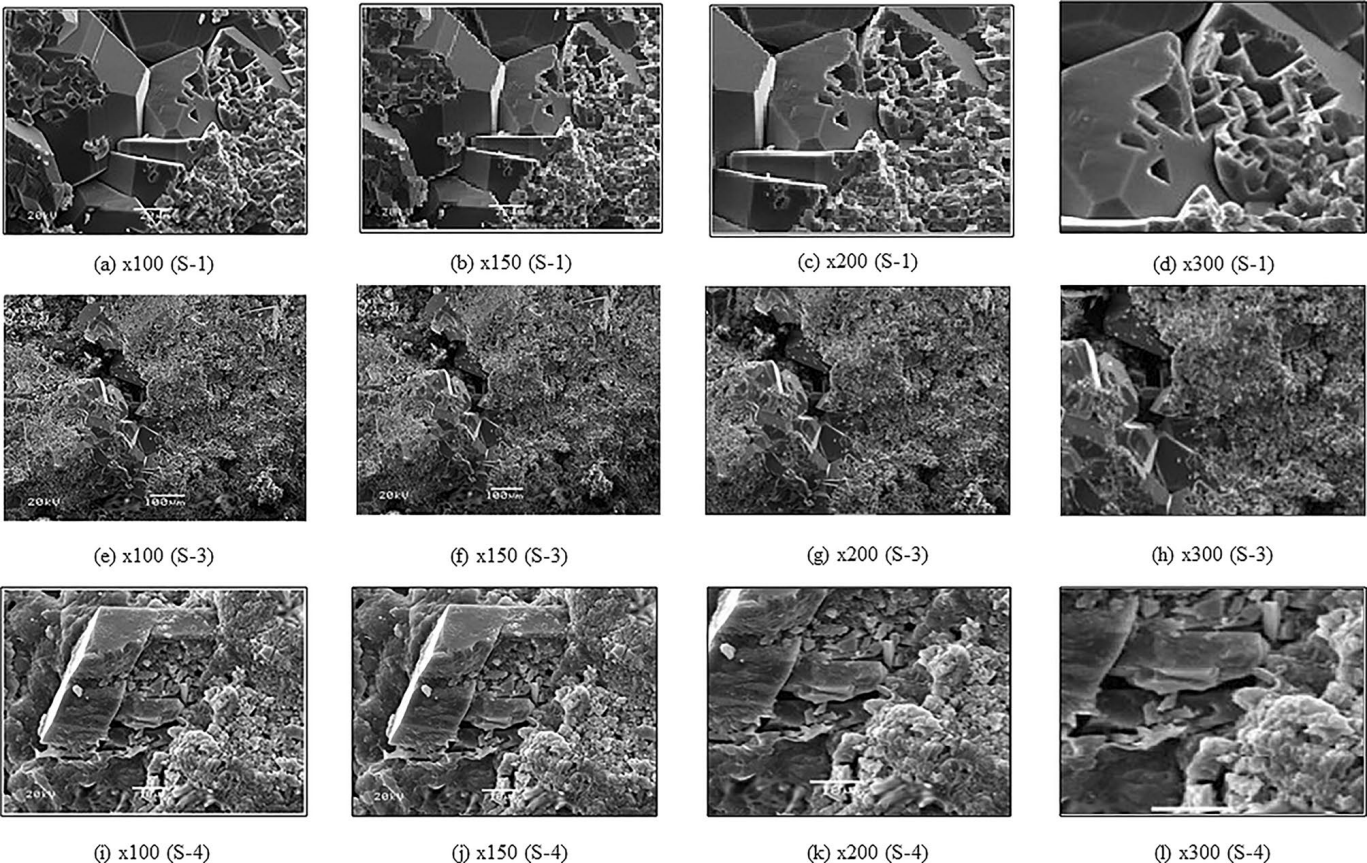
Original 2D SEM images of carbonate samples at different magnification: × 100, × 150, × 200, and × 300. (**a–d**) S-1, (**e–h**) S-3 and (**i–l**) S-4 (from left to right).

**Figure 10:**
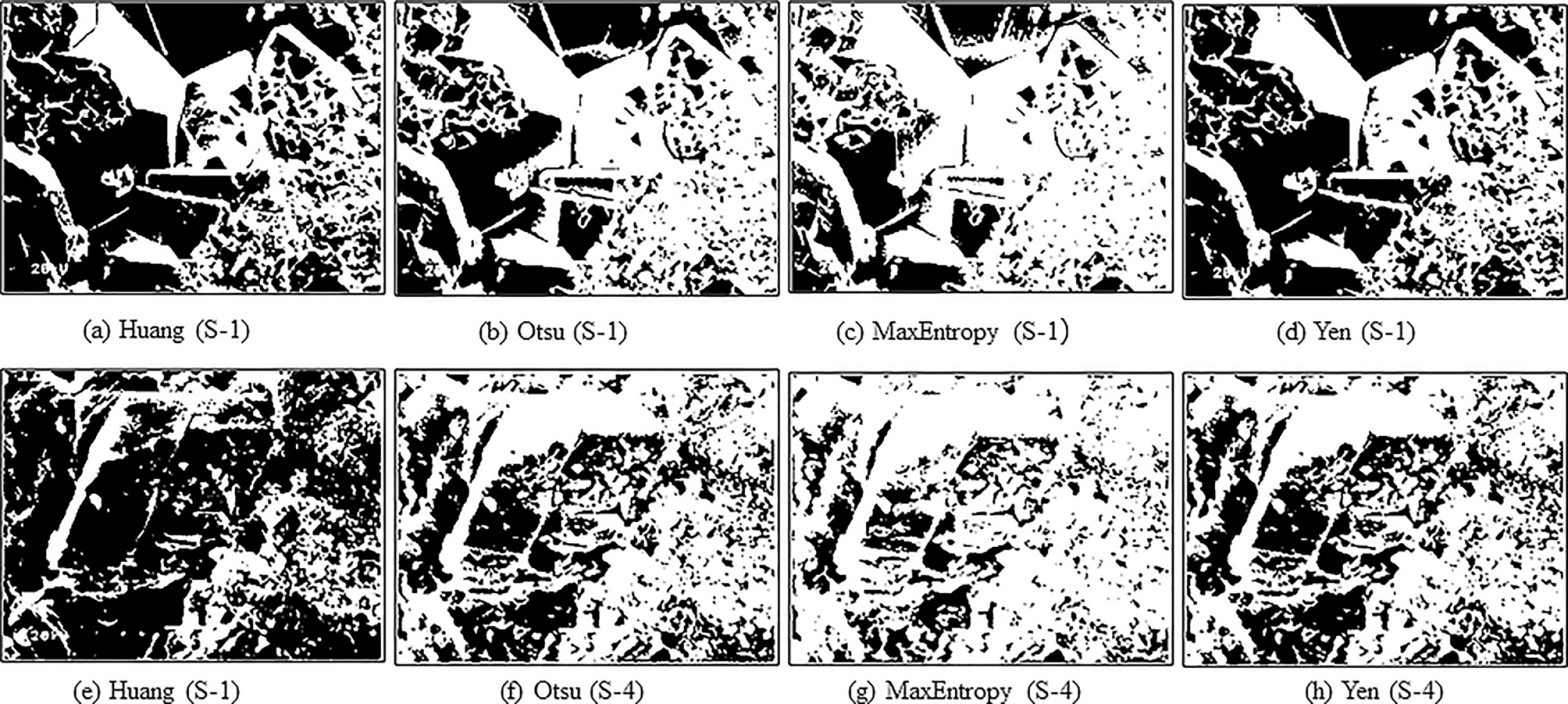
Comparison of the binary images obtained by different threshold segmentation algorithms. From left to right Huang, Otsu, MaxEntropy, and Yen algorithms. (**a–d**) S-1 and (**e–h**) S-4. The white and black portions represent the carbonate matrix and pores respectively.

The original 2D scanning electron microscopy (SEM) images of carbonate samples, referred to as S-1 from the Kuldhar member, S-3 from the Hamira member, and S-4 from the Badabag member are shown in Fig. 9 at various magnifications. Increasing the magnification reveals macroscopic cracks within the samples that have a notable impact on carbonate sample permeability. Conversely, lower magnification provides a wider field of view, showing a broader range of pore visibility. To examine the pore characteristics of the carbonate samples, we used all four available threshold algorithms for the grayscale to binary image conversion process to determine the most suitable algorithm. We conducted a gray scale-to-binary image conversion using four threshold algorithms (Huang, Otsu, MaxEntropy, and Yen) to determine the most suitable algorithm. Figure 10 shows the results of binary images, where black and white areas represent pores and the matrix of the carbonate samples, respectively. The MaxEntropy threshold algorithm shows relatively low pore occupancy compared to other threshold algorithms with pores occupying only a small percentage of the images.

**Figure 11:**
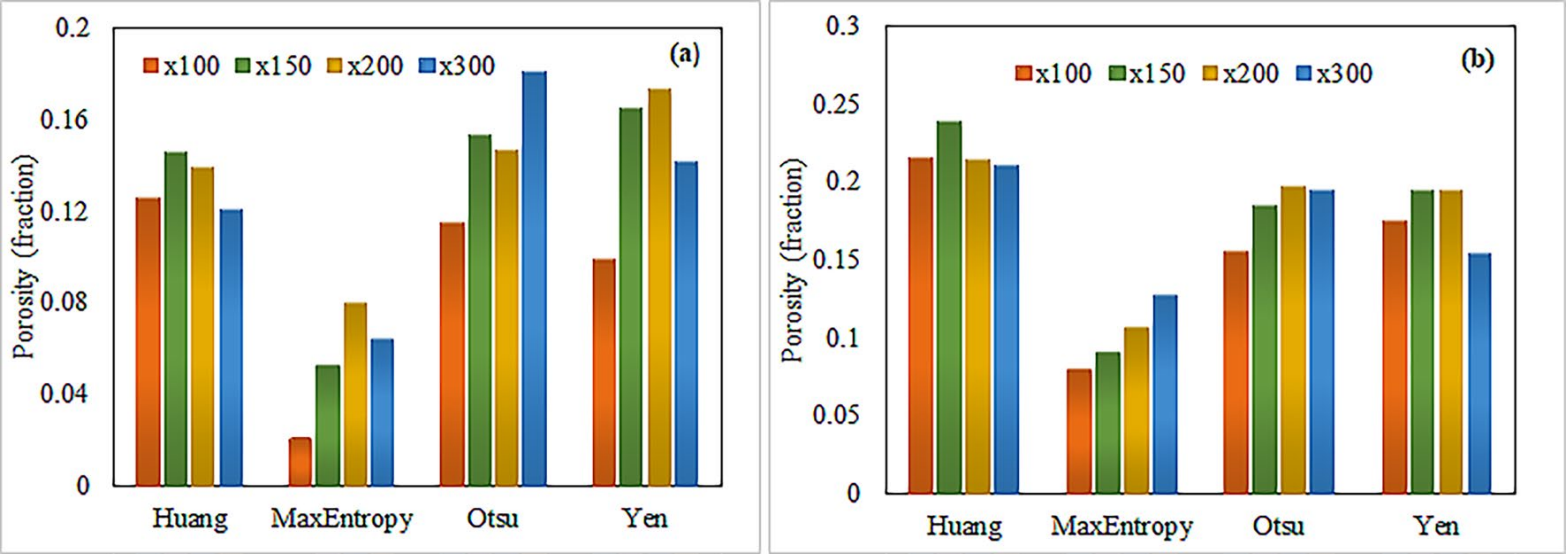
Comparison of porosity histograms of carbonate samples obtained by different threshold segmentation algorithms at various magnifications. (**a**) S-1 and (**b**) S-4.

Computing the porosity involved analyzing the binary images, counting the pore pixels, and dividing them by the total number of pixels. Figure 11 shows the porosity histograms of carbonate samples S-1 and S-4 at various magnifications (× 100, × 150, × 200, and × 300) got by calculating the grayscale threshold value by four different segmentation algorithms. We observed significant variations in the porosity values among these algorithms. The Otsu and Yen algorithms yielded porosity values ranging from 0.10 to 0.18 for S-1 and 0.14 to 0.19 for S-4. These values exceed the 10% porosity threshold, thus showing the inefficiency of these algorithms compared to petrographic studies. The Huang algorithm exhibited inconsistent behaviour in porosity calculation, resulting in varying outcomes across different samples and magnifications. At × 150 magnification, S-4 showed a maximum porosity of over 0.24, while S-1 had a minimum porosity of 0.12 at × 100 magnification. The Huang algorithm’s limited robustness can be the reason for these disparities.

**Figure 12:**
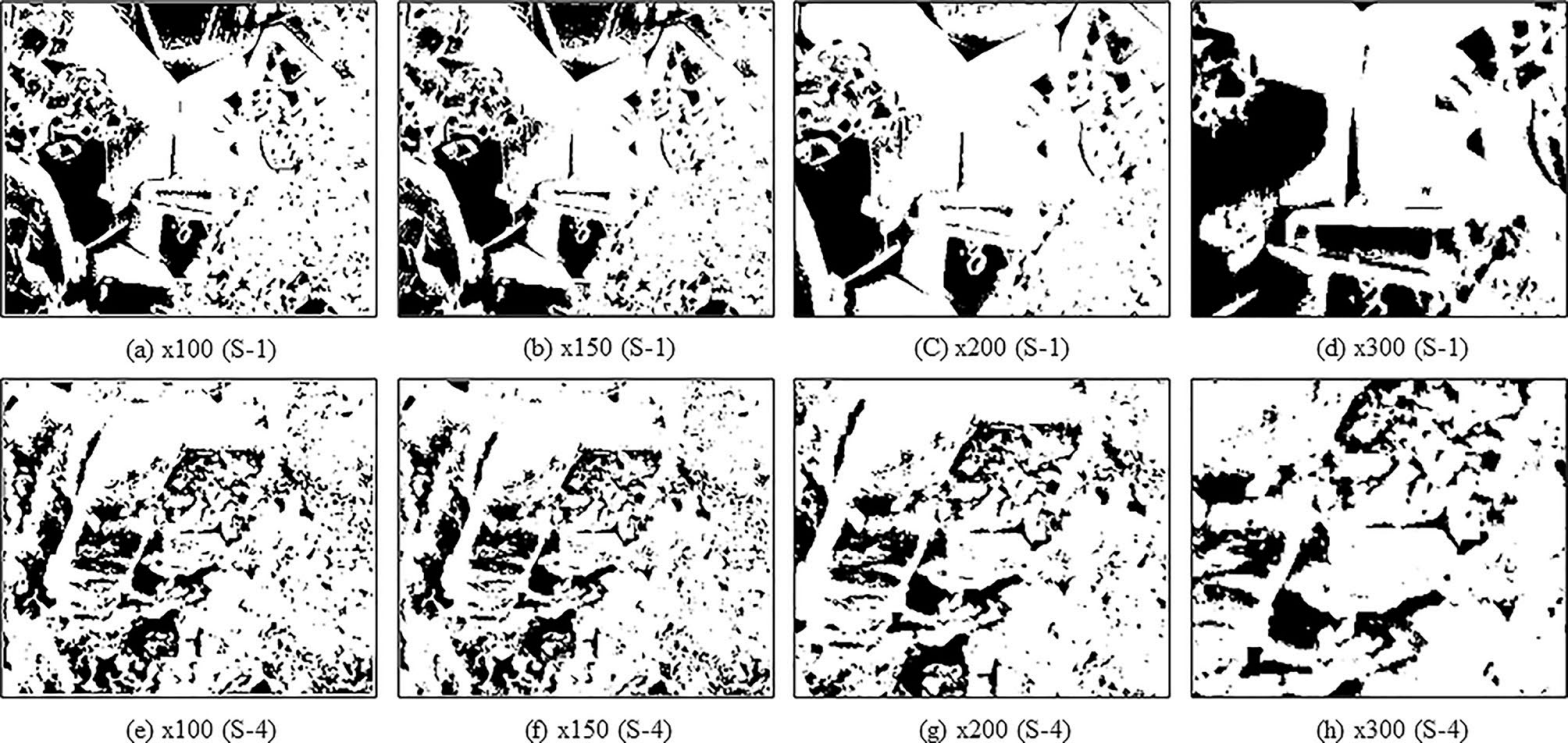
Binary segmented images of carbonate samples (S-1 and S-4) segmented by the MaxEntropy algorithm at various magnifications (Left to Right × 100, × 150, × 200 and × 300).

In contrast, the MaxEntropy algorithm consistently produced porosity values below 0.12 for all samples and magnifications. Specifically, the values are ranging from 0.02 to 0.08 for S-1 and 0.07 to 0.12 for S-4. To validate the accuracy of the threshold segmentation algorithm, we compared the obtained porosity results with measured porosities from petrographic investigations^63^ (see Table 3), which reported porosity ranges of 0.10 to 0.12 for S-1 and 0.08 to 0.10 for S-4. Overall, the threshold segmentation based on the MaxEntropy algorithm is more reasonable, as it generates porosities for carbonate samples that are closer to the findings of petrographic studies. We identified the MaxEntropy algorithm-generated binary images for further analysis in this study. Figure 12 depicts the binary images generated by the MaxEntropy algorithm, which correspond to the original SEM images of S-1 and S-4 shown in Fig. 9. These binary images were utilized to determine the various pore network parameters, including pore size distribution (PSD), throat radius, grain size distribution (GSD) and 2D coordination number and estimate LBM based permeability for computation of machine learning (ML) algorithms.

**Table 3:** Comparison of porosity calculated by MaxEntropy algorithm with petrographic studies.

Sample		Porosity(fraction)	
Label	Magnification	MaxEntropy	Petrographic studies
S1	× 100	0.021	0.108
	× 150	0.052	
	× 200	0.08	
	× 300	0.064	
S4	× 100	0.079	0.125
	× 150	0.09	
	× 200	0.107	
	× 300	0.127

### Determination of pore network parameters

**Figure 13:**
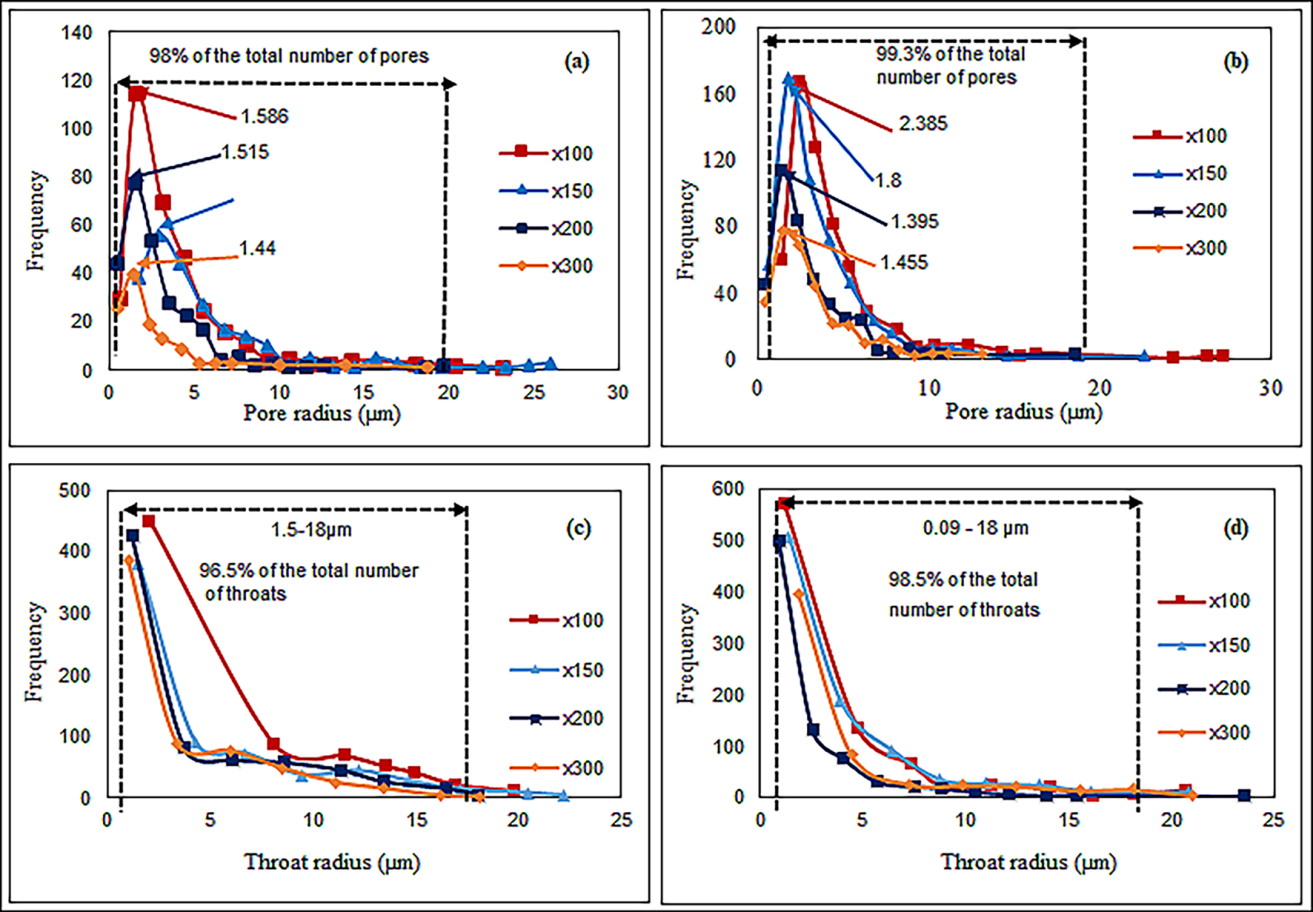
Pore radius and throat radius distribution of carbonate sample with frequency at various magnifications. Pore radius distribution: (**a**) Sample S-1, (**b**) Sample S-4. Throat radius distribution: (**c**) Sample S-1, (**d**) Sample S-4.

We effectively examined pore characteristics at the micrometer level by applying the watershed algorithm mentioned in this section = to determine pore network parameters. Figure 13a,b display the pore size distribution variations in carbonate samples S-1 and S-4, observed at magnifications of × 100, × 150, × 200, and × 300, respectively. These plots depict normal distribution curves representing the frequencies of different pore sizes. It is observed that smaller pores are more significantly affected by magnification compared to larger ones. In this study, the smallest detectable pores for S-1 and S-4 have a pore radius of less than 0.48 µm, regardless of magnification. Across all carbonate samples, there is a higher proportion of small-sized pores and a lower proportion of larger pores. The percentage of pores decreases as pore size increases, with larger pores representing only a small fraction of the total. Overall, over 98% of the observed pores in all carbonate samples fall within the range of 0.44 to 20 µm. It is important to note that lower magnification provides a broader range of pore sizes, but some smaller pores may remain undetected. While higher magnification reveals only a portion of the smaller micro-pores, emphasizing the significance of magnification in image analysis. These observations hold value for studying pore structure characterization^4^.

The distribution of throat radius plays a crucial role in determining permeability and controlling fluid flow in carbonate reservoirs. Figure 13c,d illustrate the variation in throat radius for carbonate samples S-1 and S-4 at different magnifications. The peak throat radius shows a relatively narrow distribution, falling within the range of 0.9 to 2.04 µm, and it noticeably diminishes as magnification levels rise. There is an inverse relationship between throat radius and magnification, with an increase in magnification leading to a decrease in the number of throats. Sample S-4 exhibits a greater number of throats with a smaller radius compared to sample S-1. When the throat radius is less than 2 µm, the number of throats increases as magnification decreases, while the number of throats decreases with an increase in the throat radius. Only 2 to 4% of the total number of throats have a radius greater than 18 µm. The study reveals that it predominantly distributed the throat radius in carbonate samples within a range of less than 18 µm. The influence of magnification is more prominent for smaller throat radius but diminishes as the throat radius increases. For a larger throat radius, the curves overlap regardless of magnification.

**Figure 14:**
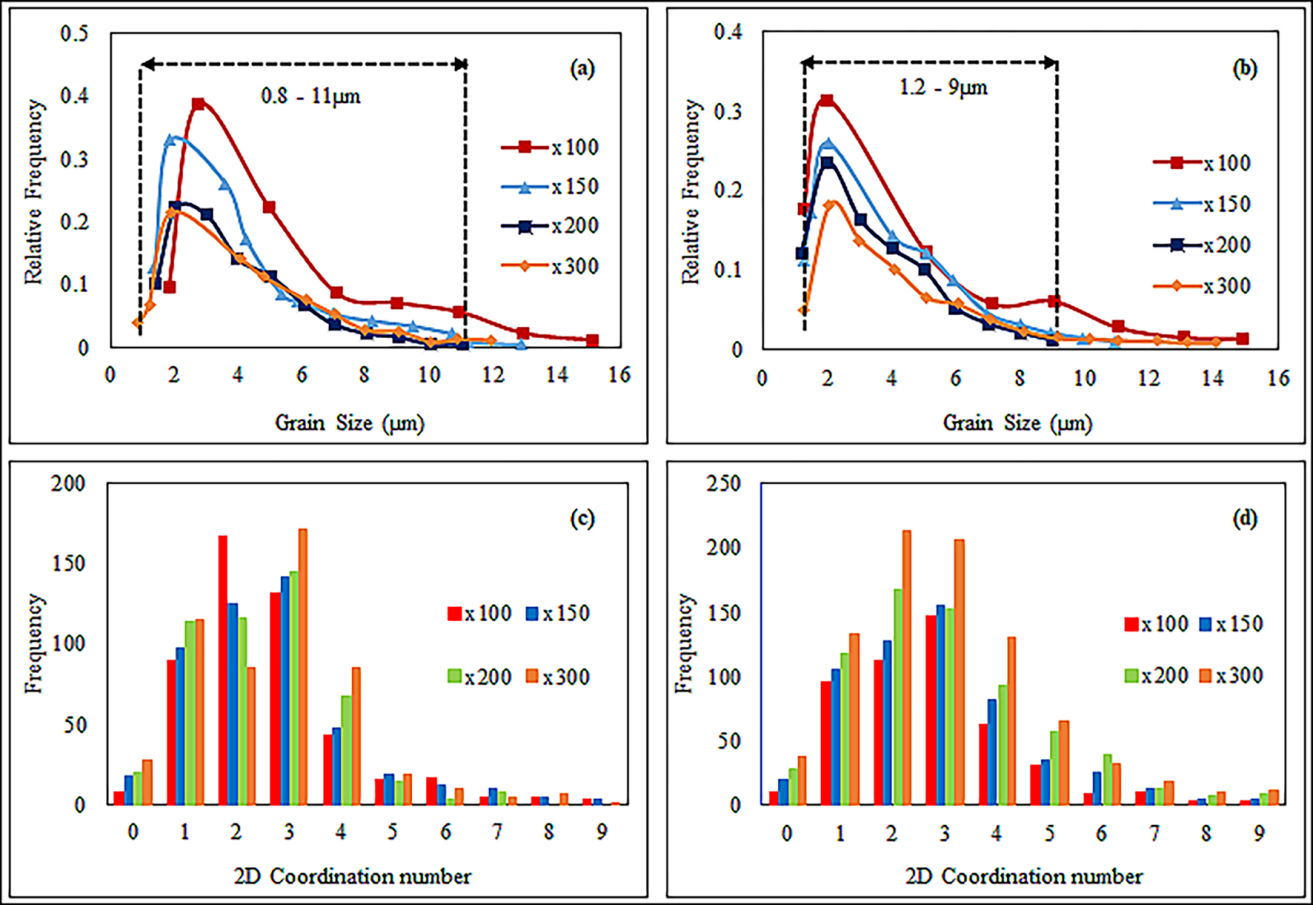
Grain size and coordination number distribution of carbonate samples with relative frequency at various magnifications. Grain size distribution: (**a**) Sample S-1, (**b**) Sample S-4. Coordination number distribution: (**c**) Sample S-1, (**d**) Sample S-4.

We analyzed the grain size distribution of carbonate samples S-1 and S-4 by using 2D SEM images at various magnifications. Figure 14a,b depict the variation of grain size distribution at different magnification. The study revealed a limited range of grain sizes, predominantly between 0.8 and 10 µm. Initially, the number of grains increased with grain radius up to 2 µm, after which it decreased. The results showed that the peak radius of sample S-1 was greater than 2 µm at lower magnification but decreased as magnification increased. The impact of magnification was more noticeable for smaller grain sizes and became less significant as the grain radius increased. We observed that lower magnification did not reveal a higher number of grains compared to higher magnifications for the carbonate samples.

Figure 14c,d depict the frequency distribution of carbonate samples S-1 and S-4 at different magnifications, along with their corresponding computed 2D coordination numbers. As magnification increases, pore connectivity within both samples improves significantly. Higher magnification allows for the detection of even the smallest micro throats in the porous media, some less than 0.09 µm in size. As a result, the coordination number at different magnifications reflects varying levels of pore connectivity in the carbonate samples. Lower magnifications reveal larger throats, showing lower pore connectivity, while higher magnification enhances pore connectivity across all samples. The highest pore connectivity occurs within the coordination number range of 1 to 4, and pore connectivity decreases as the coordination number increases. Finally, we observed that the magnification relationship profoundly affects the behaviour of the carbonate reservoir by influencing the pore network parameters. These parameters impact the permeability of porous media. Higher magnification reveals the true intricacies of pore structure, influencing pore size, shape, and connectivity. A well-connected network of larger pores system shows results in the higher permeability.

### Permeability calculation using LBM

We estimated the throat permeability of carbonate samples using the lattice Boltzmann method (LBM) simulation. The lattice Boltzmann method (LBM) simulation used a pressure differential assumption across the pore network and determined the pressure through the center of the pores. The fluid flow continuity equation is applied to each pore body to describe the steady-state process. This study only considered a single-phase, incompressible fluid. By applying a linear system of equations, we calculated the pressure of the pore bodies whereas the total flow rate was then computed using the determined pressure. Finally, Darcy’s law was used to calculate the total permeability of the pore network in each carbonate sample.

**Figure 15:**
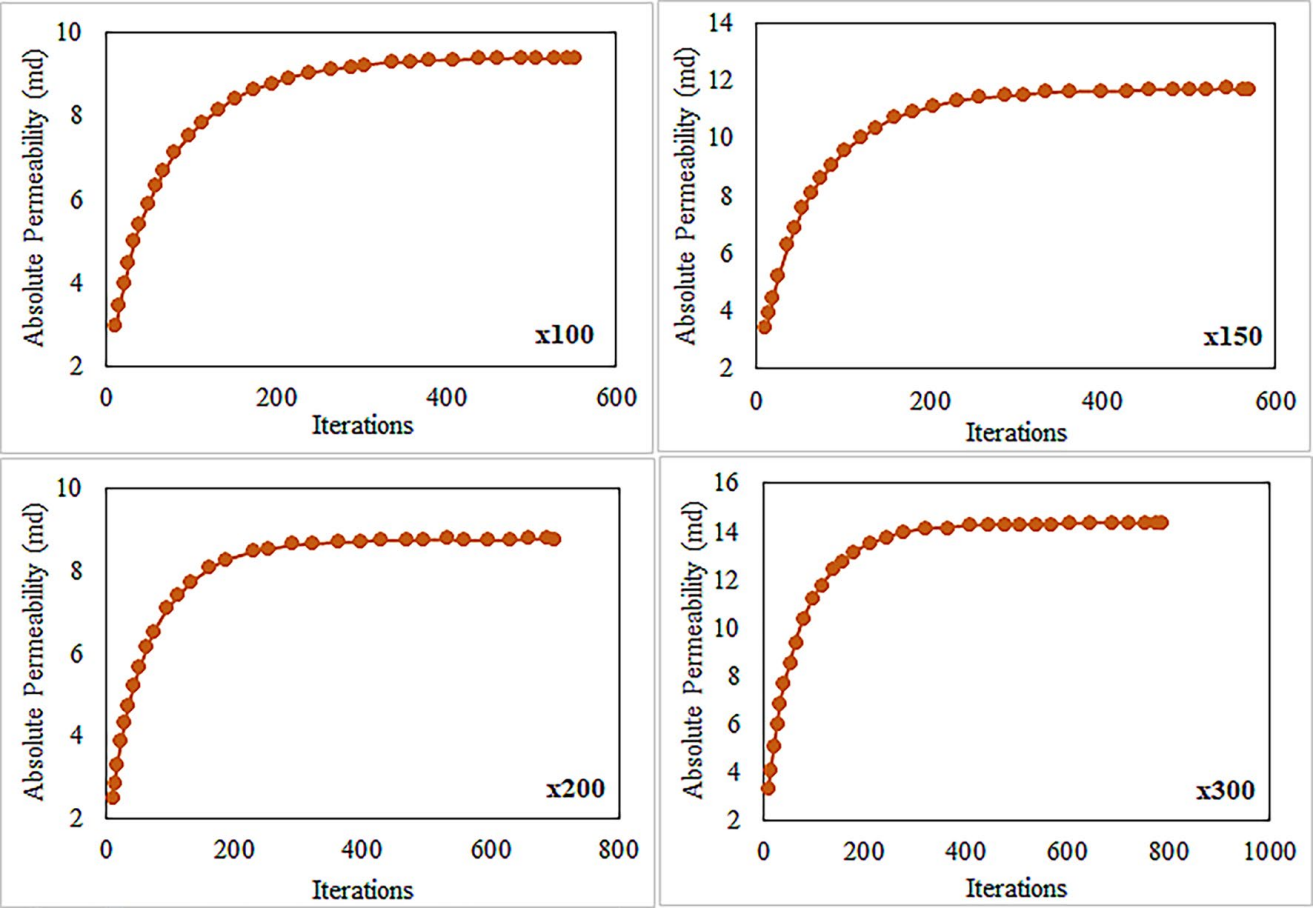
The measured permeability of sample S-1 at various magnifications with number of iterations was obtained by LBM simulation approach.

**Figure 16:**
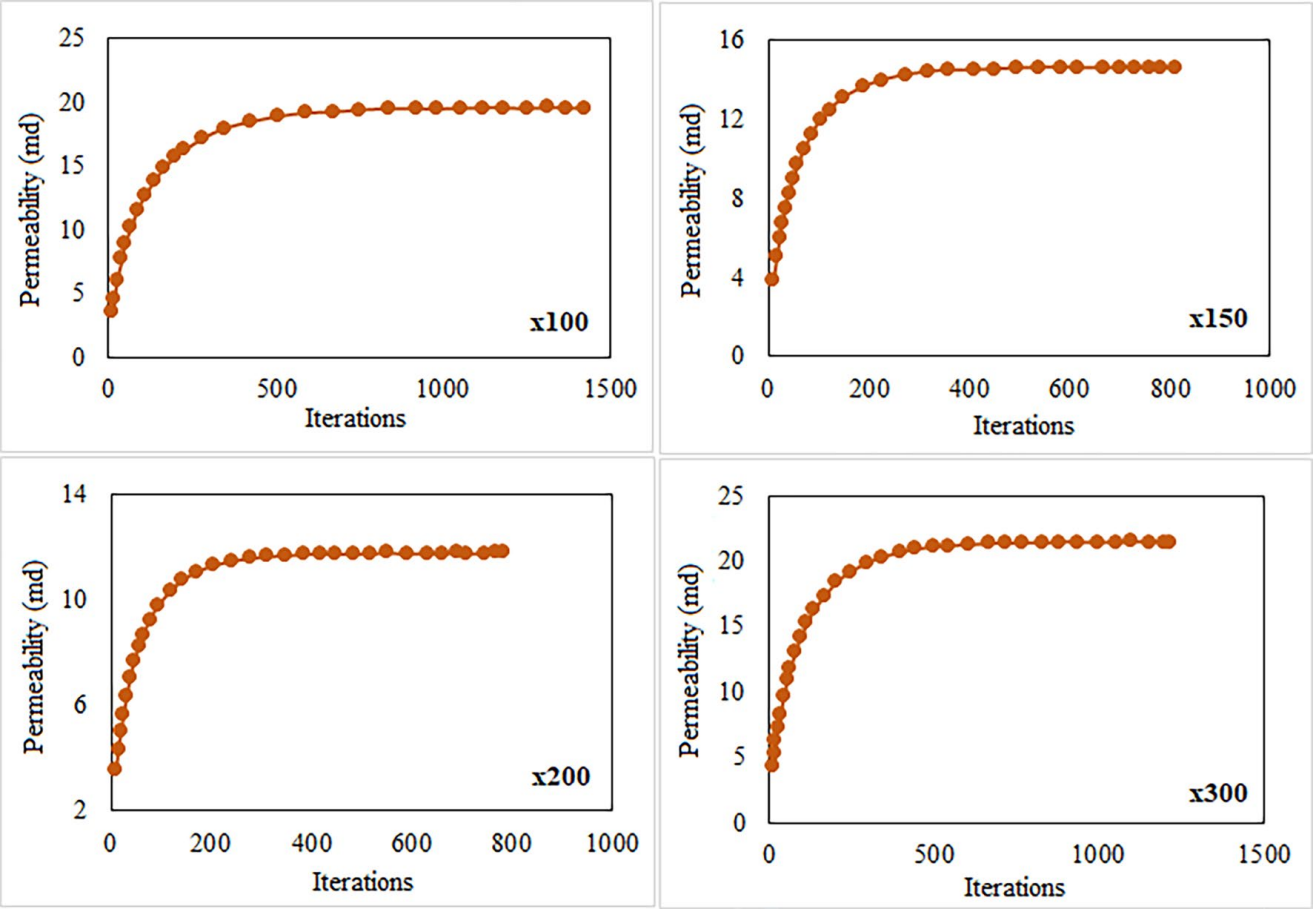
The measured permeability of sample S-4 at various magnifications with number of iterations was obtained by LBM simulation approach.

**Figure 17:**
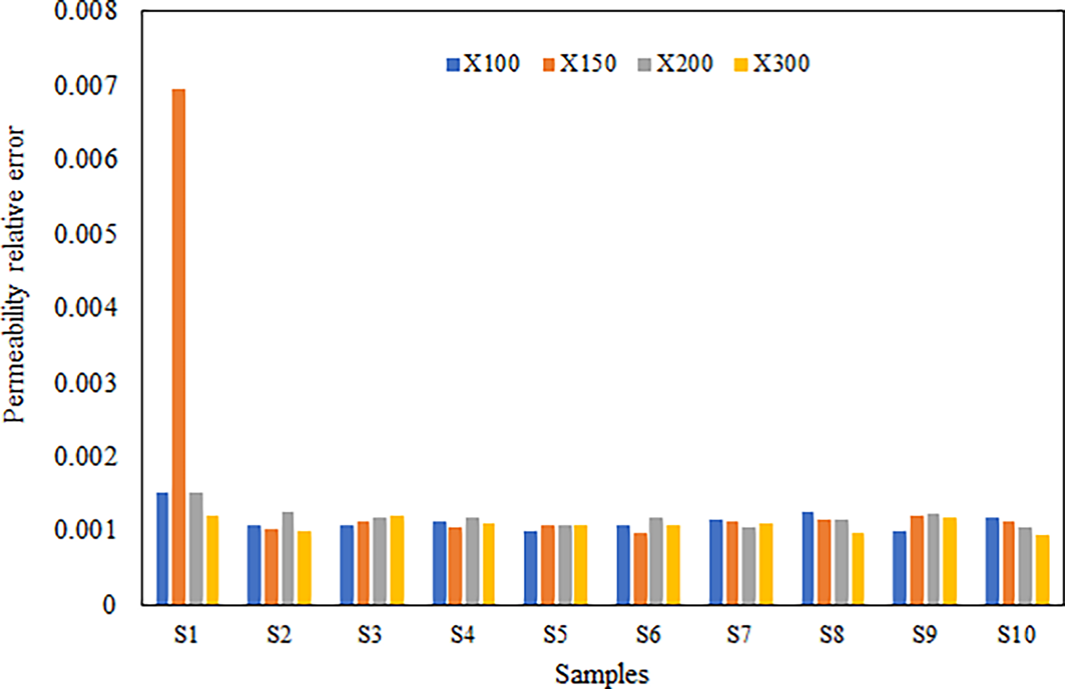
Illustrates a histogram depicting the distribution of relative error values for numerical simulation results of permeability across all samples at various magnifications.

**Figure 18:**
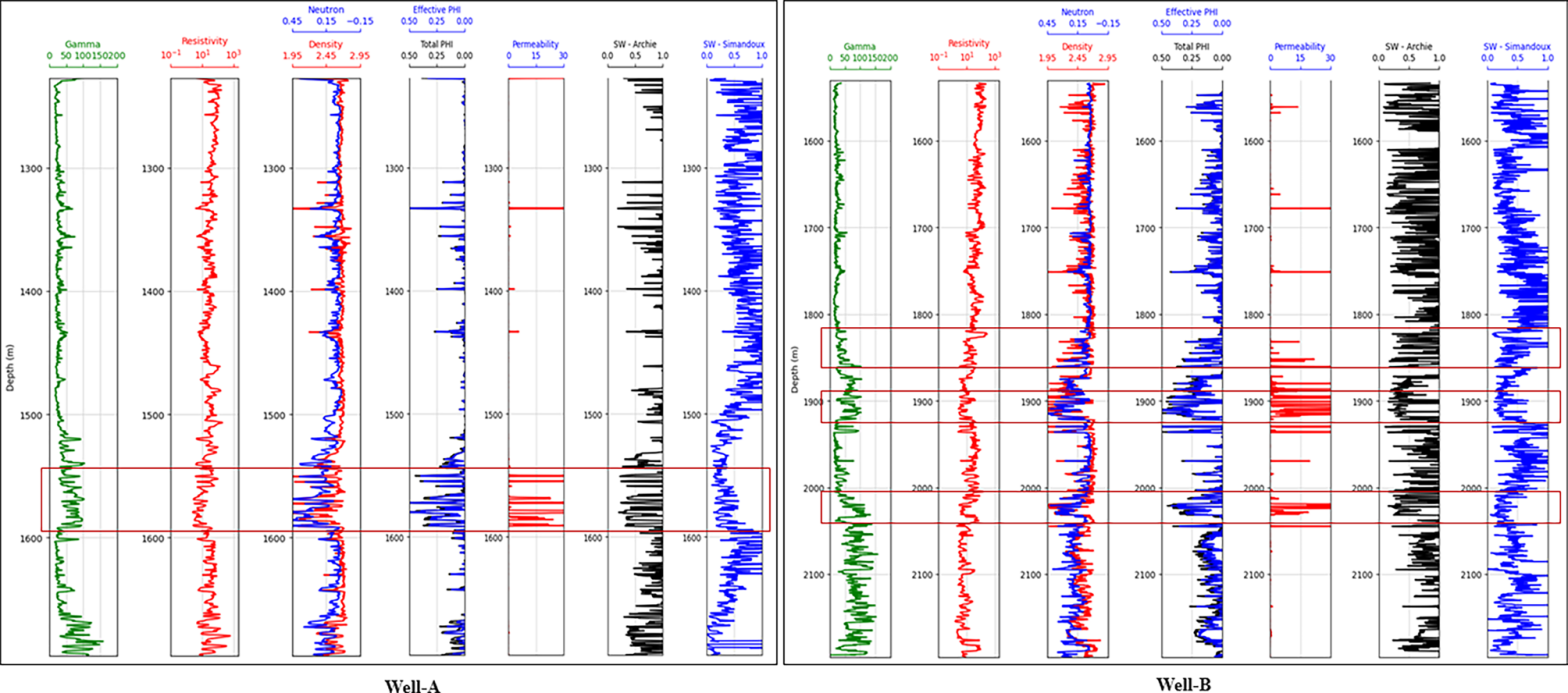
Calculated permeability and water saturation beside of conventional well logs for Well-A and Well-B. The marked regions represented by the study area.

The permeability of carbonate samples was determined through Lattice Boltzmann Method (LBM) simulations, which achieved iterations convergence. Figures 15 and 16 show convergence curves at different magnifications for samples S-1 and S-4, respectively based on results of LBM simulation. Once the simulation reached equilibrium, the samples’ permeability remained constant even after conducting further iterations. We observed that the LBM permeability of both S-1 and S-4 increased with the number of iterations until a specific value was reached, beyond which it remained constant regardless of magnification. The calculated LBM permeability increased with magnification for most samples. The total permeability values of carbonate samples at various magnifications ranged from 0.92 to 21.42 millidarcies (md), as shown in Table 4. It was observed that the impact of magnification on permeability varies among different samples. In some samples, permeability increases with magnification, while in others, it decreases. This variability can be attributed to rock matrix and presence of smaller pores at higher magnifications. We have computed the relative error values for the numerical simulation of permeability results as illustrated in Fig. 17. These results consistently exhibit low relative errors across all carbonate samples, signifies a close alignment between numerical simulation LBM permeabilities and the measured permeabilities from well logs in the same geographical area. The average permeability values got from well-A and well-B are 10.041md, and 15.561md, respectively. Meanwhile, the average permeabilities simulated through LBM are: 5.418md at × 100 magnification, 7.55md at × 150 magnification, 10.13md at × 200 magnification, and 13.588md at × 300 magnification. These simulation results closely align with the empirically calculated permeabilities, particularly at higher magnification levels. Figure 18 illustrates the empirical permeability calculation got from well logs, which serves to validate the accuracy of the LBM permeability estimations.

**Table 4:** The measured permeabilities of carbonate samples at various magnifications obtained by LBM simulation technique.

Sample	Measured LBM* permeability (md)			
	× 100	× 150	× 200	× 300
S1	9.35	11.71	8.73	14.33
S2	4.71	7.021	9.06	12.62
S3	8.70	9.15	14.65	16.88
S4	11.80	14.59	19.41	21.42
S5	8.19	11.25	17.46	20.41
S6	5.13	6.35	8.73	9.18
S7	1.95	6.13	10.14	15.72
S8	0.92	1.68	1.97	4.31
S9	2.25	4.93	7.32	14.8
S10	1.18	2.72	2.66	6.18

*Lattice Boltzmann method.

### Permeability prediction using machine learning (ML)

This study aimed to explore the predictive capabilities of machine learning (ML) models, specifically Support Vector Machines (SVM) and Artificial Neural Networks (ANN), for estimating the permeability of carbonate samples. Five pore network parameters, including porosity, average pore radius, average throat radius, average grain size, and average coordination number, were selected as input features, while the calculated LBM permeability values served as the target output. To ensure reliable ML model performance, high-quality data were essential. Therefore, the dataset was divided into three subsets with 70% allocated for training, 15% for testing, and the remaining 15% for validation purposes. The dataset contained a total of 200 data points, we utilized 140 data points for training, 30 data points for testing, and 30 data points for validation for our ANN and SVM models. Table 5 shows the statistical analysis of the data. To determine the most accurate ML model for permeability prediction, we trained and evaluated the models and used model evaluation metrics.

**Table 5:** A statistical description of input data used for machine learning prediction.

Input parameter	Min.	Max.	Mean	Range	SD	Kurtosis	Skewness
Avg. throat radius	1.894	30.24	8.179	28.346	6.116	2.749	1.575
Avg. pore radius	2.223	24.246	9.811	22.023	5.071	0.282	0.667
Avg. grain size	2.143	16.248	7.726	14.105	2.851	0.809	0.706
Avg. coordination number	1.302	4.743	2.635	3.441	0.957	− 0.928	0.363
Porosity	0.008	0.229	0.099	0.221	0.065	− 0.871	0.438
Permeability (LBM)	0.92	21.42	8.992	20.5	5.675	− 0.704	0.485

**Figure 19:**
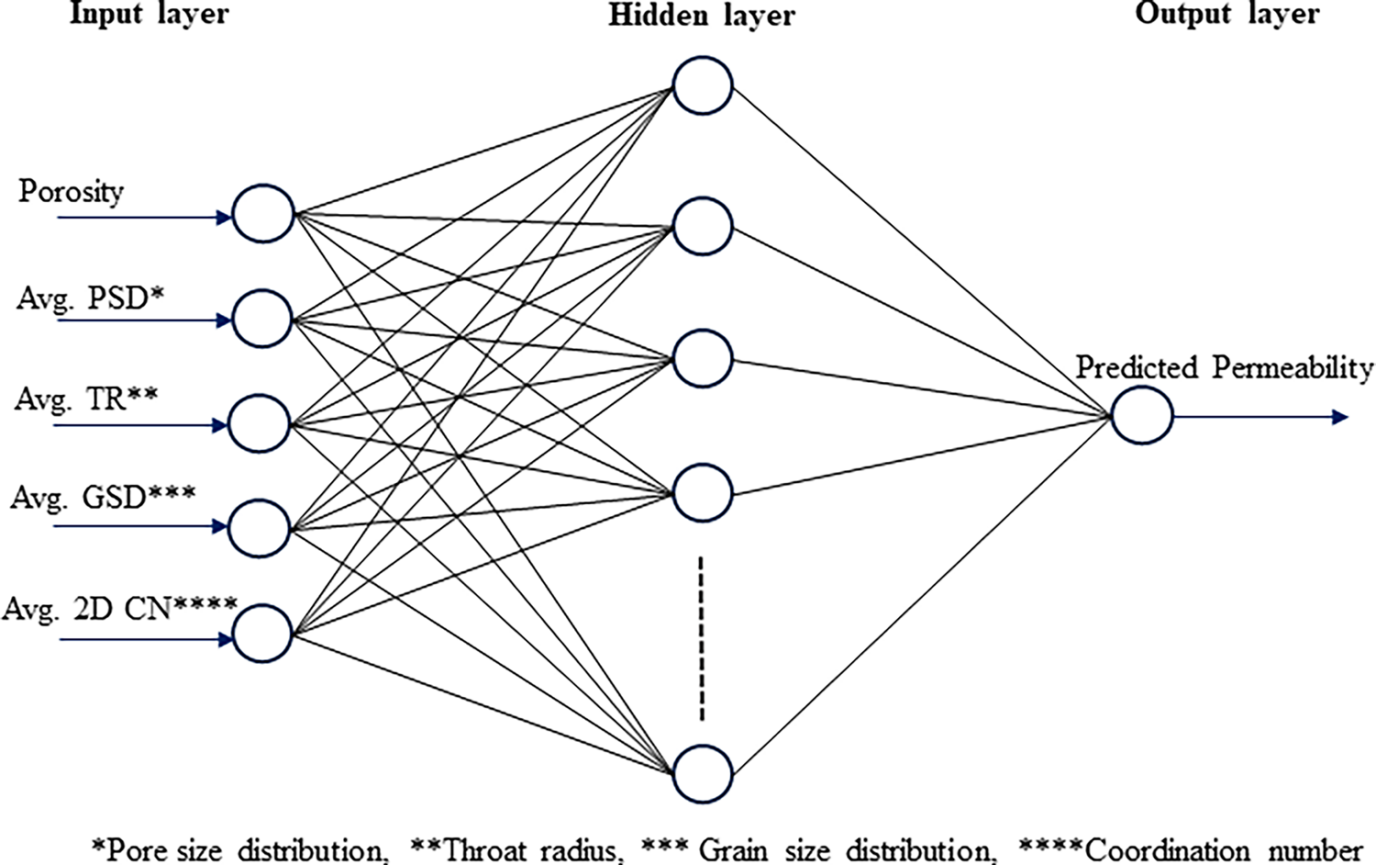
Schematic representation of ANN for permeability prediction with one input layer (5 input nodes), one hidden layer (10 nodes) and one output layer (one node).

**Figure 20:**
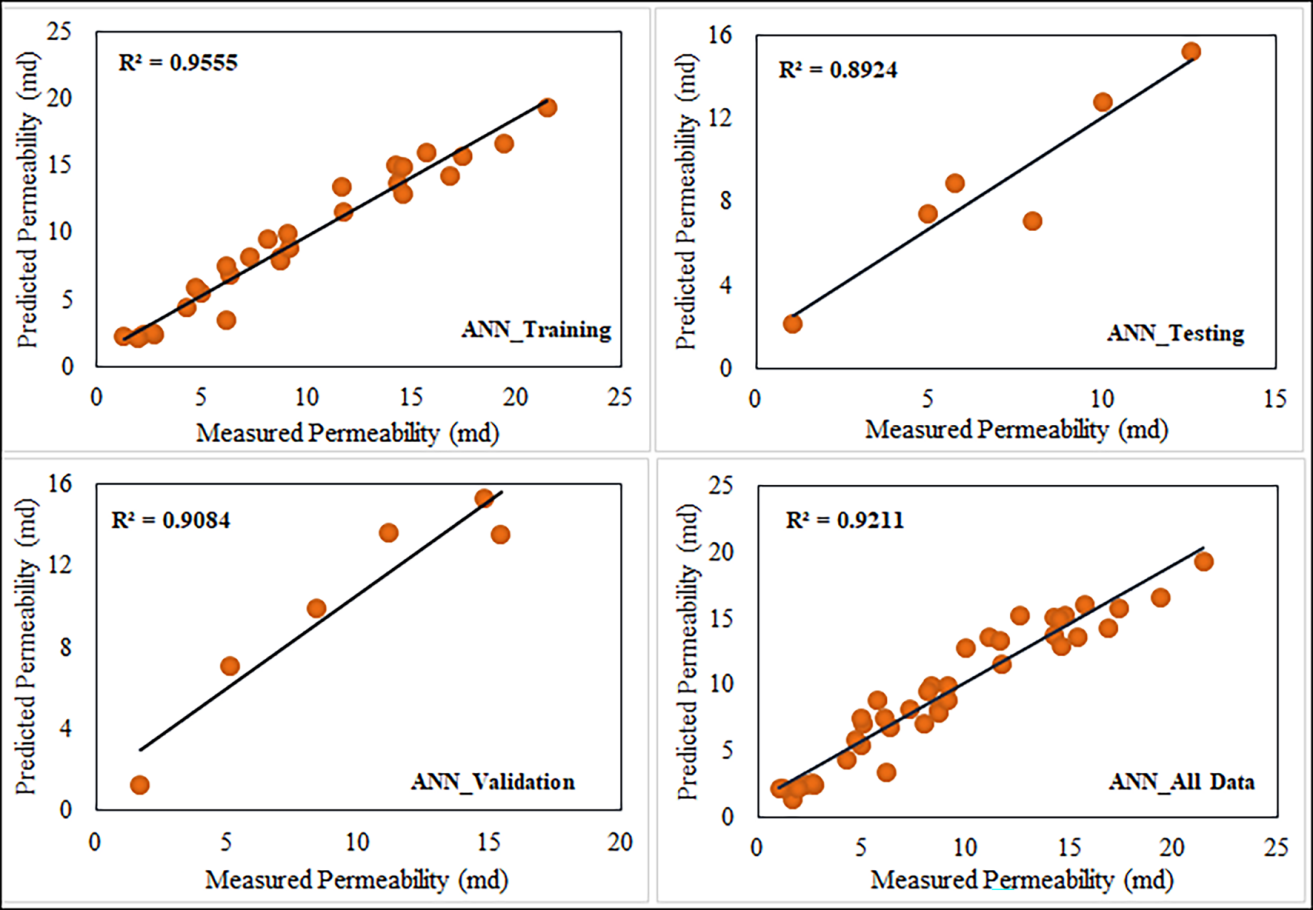
Comparison of ${R}^{2}$ cross plots between measured and predicted permeability using Artificial Neural Network (ANN) at different datasets.

**Figure 21:**
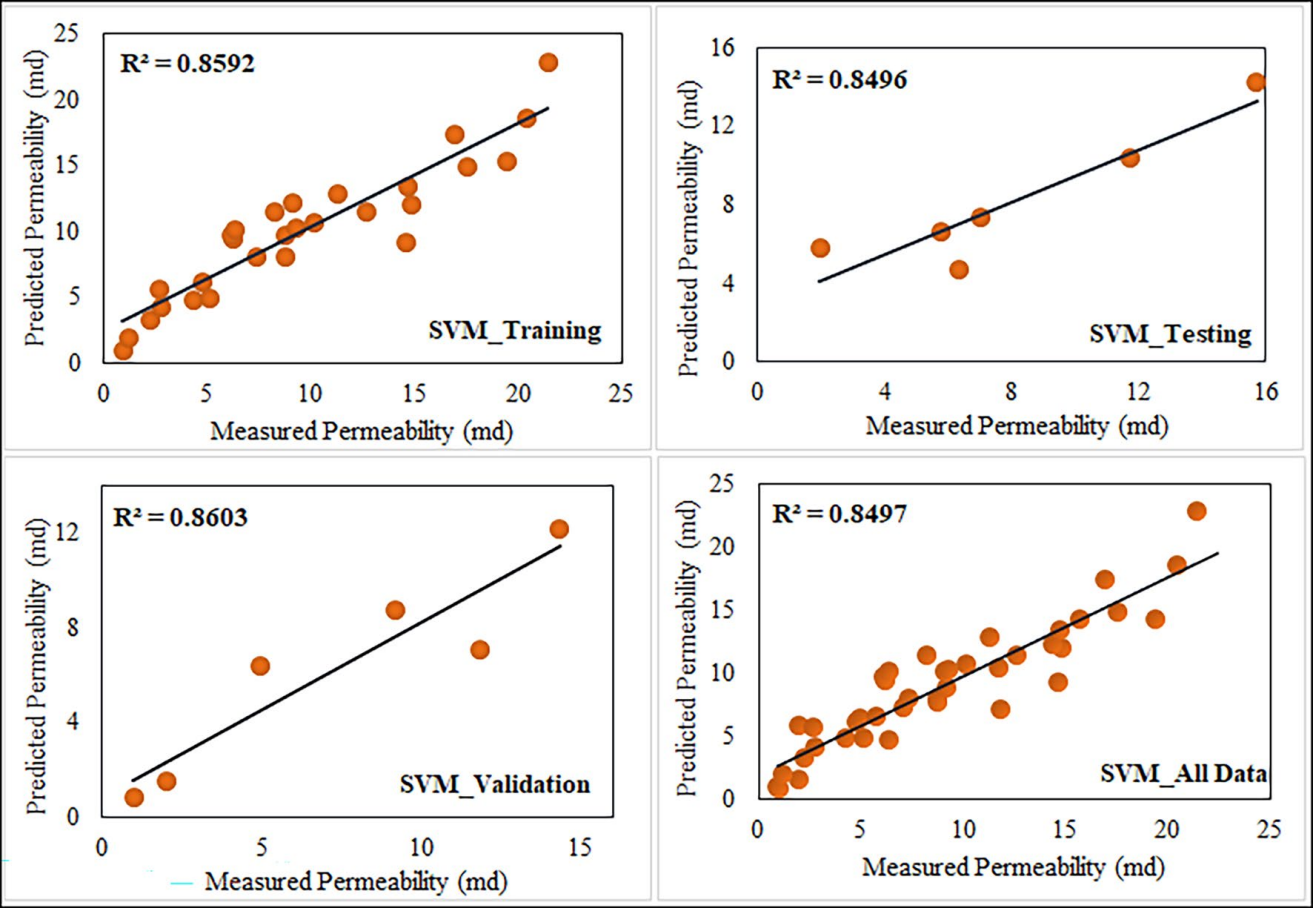
Comparison of ${R}^{2}$ cross plots between measured and predicted permeability using Support Vector Machine (SVM) at different datasets.

**Figure 22:**
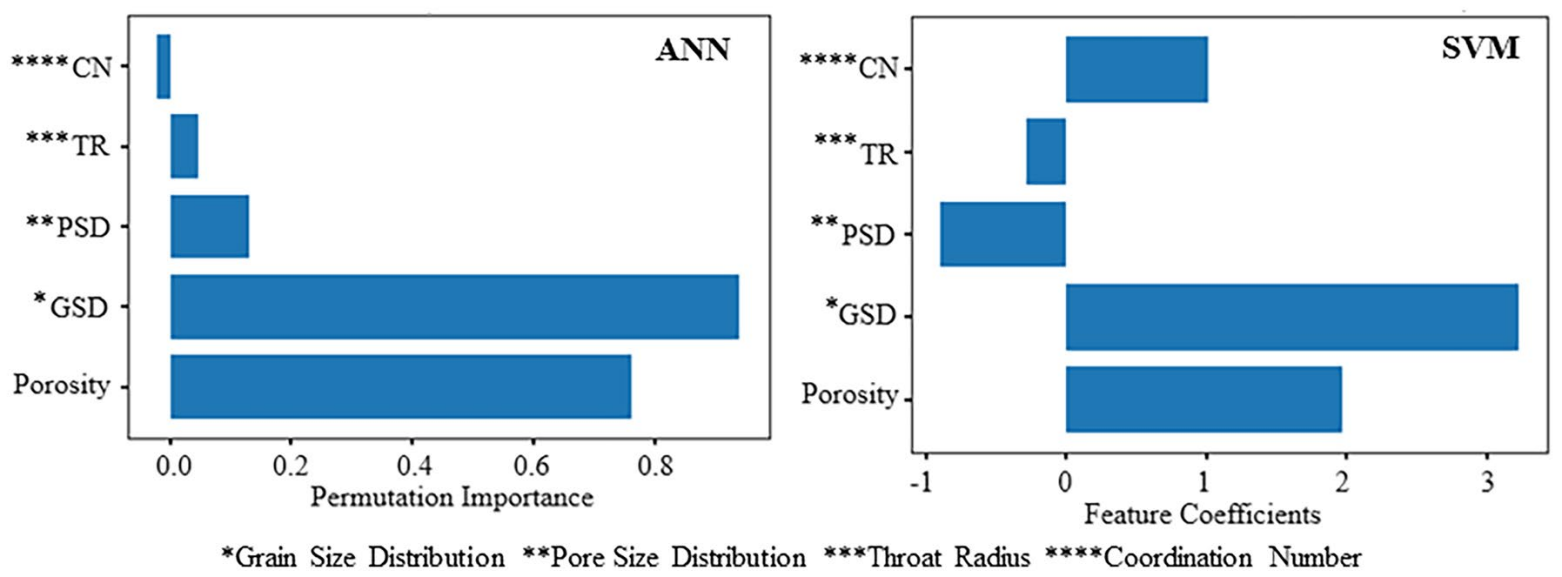
Variable importance of features included in machine learning algorithms for permeability prediction.

The analysis of estimating the permeability yielded a neural network architecture with 10 neurons for the ANN model. We implemented the input and hidden layers with a linear-type activation function, while the hidden and output layers used a TAN-sigmoidal type activation function. Figure 19 illustrates the visual representation of the several steps taken by the ANN model to estimate the permeability. Figure 20 displays the outcomes of the permeability prediction using the ANN model compared to the measured permeability values for different datasets, including training, testing, validation, and all data. The coefficient of determination (${R}^{2}$) was employed to assess the performance of the ANN model in predicting the permeability. The ${R}^{2}$ values obtained for the training, testing validation, and all data were 0.955, 0.892, 0.908, and 0.921 respectively. These results show a strong correlation between the predicted and measured permeability values. Similarly, the SVM method was utilized with linear kernel function for permeability prediction, and the ${R}^{2}$ values for training, testing, validation and all data subsets were determined as 0.859, 0.849, 0.869, and 0.849 respectively. Figure 21 shows the cross plots of predicted versus measured permeabilities using the SVM method with corresponding ${R}^{2}$ values. The obtained ${R}^{2}$ values indicate a satisfactory level of accuracy and consistency in the prediction of permeability using both ANN and SVM models. The feature importance (Fig. 22) showed that the grain size distribution (GSD) is the most influential feature, and followed by porosity in both models, underlining their significance in determining model predictions. On the other hand, coordination number (CN) exhibits the least influence on the ANN model, while throat radius (TR) has the least impact on the SVM model. Pore size distribution consistently influences both models, contributing to their predictive capabilities.

**Figure 23:**
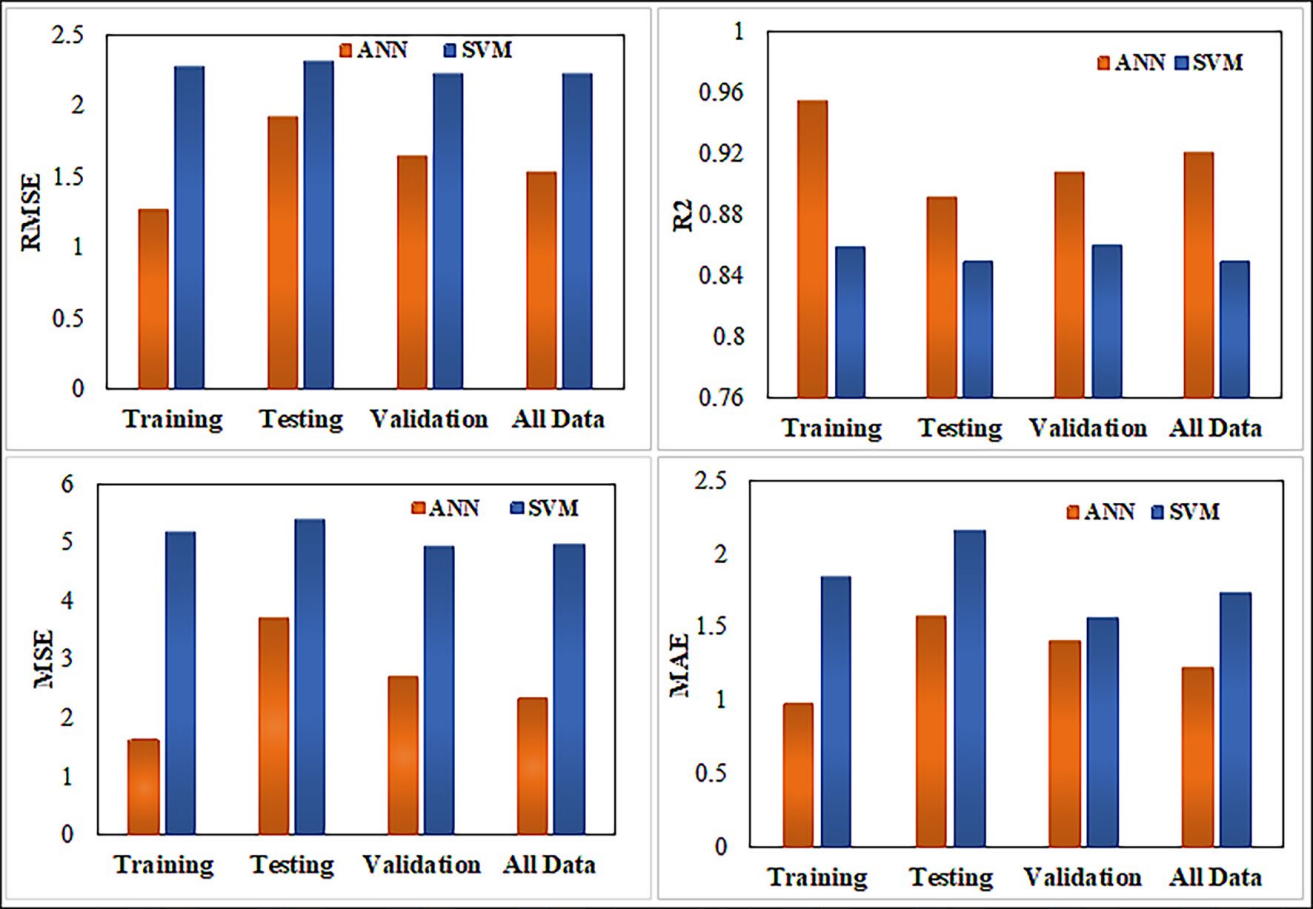
Comparison of different error metrics and ${R}^{2}$ values of predictive models of both Artificial Neural Network (ANN) and Support Vector Machine (SVM).

**Figure 24:**
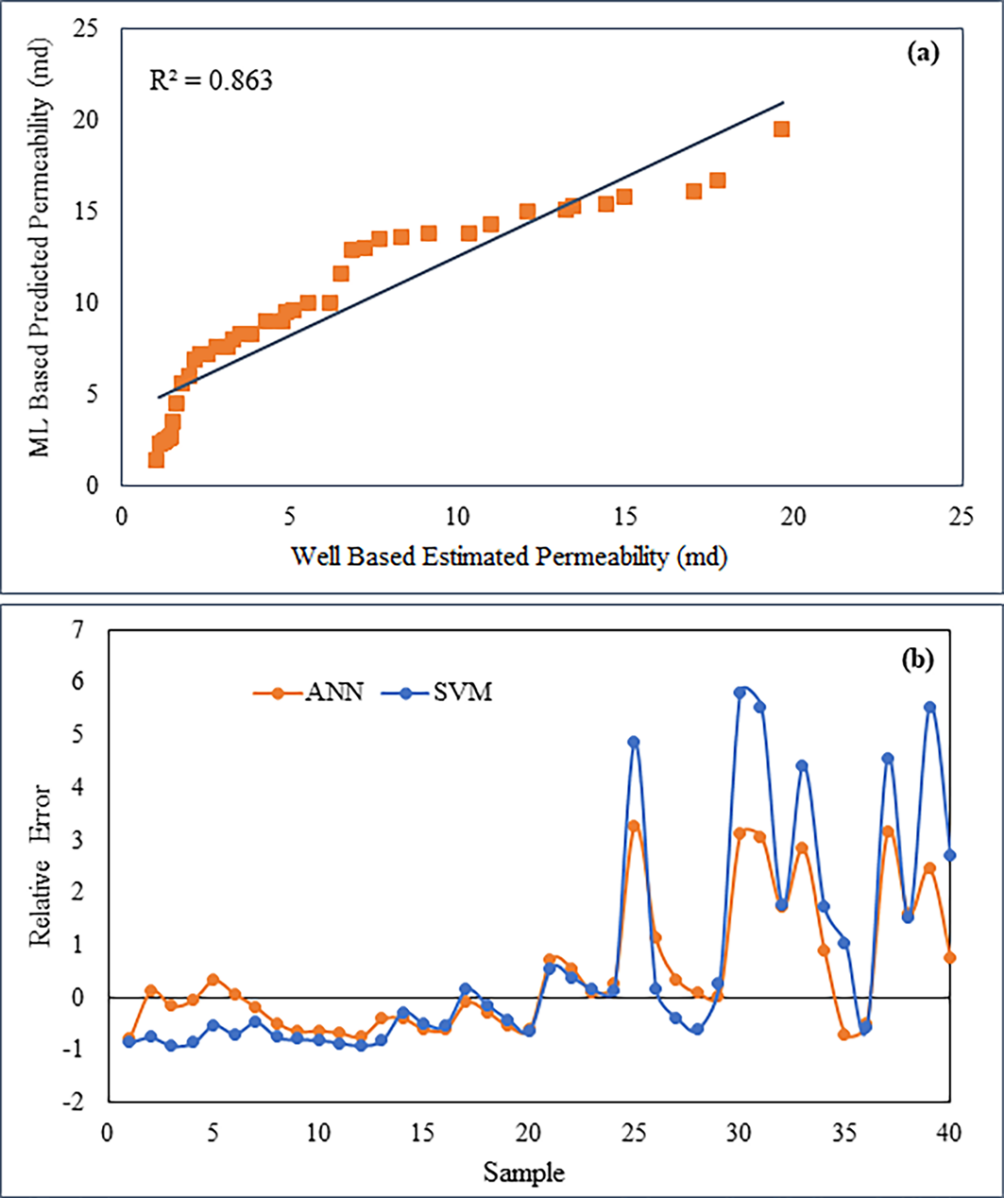
(**a**) Comparison between the permeability values predicted by our ANN model and the well-log derived permeability values specifically from Well-B within one of our study zones. This comparison serves as a crucial step in conforming the accuracy and reliability of our ANN results. (**b**) Illustrates a comparison of error results between ANN and SVM models for predicted permeability.

In order to identify the accuracy of the model is high when the $${R}^{2}$$ value approaches 1, showing a strong correlation between predicted and measured values. When the error values are close to zero, it shows that the model prediction is very close to the true values. In this study, using the result obtained for the coefficient of determination ($${R}^{2})$$ and RMSE, MSE, and MAE values (Table 6). We investigated the efficiency of the ANN and SVM models and selected the appropriate model for permeability prediction. Table 6 shows predictive models for training, testing, validation, and all data have derived the performance indices. Figure 23 shows the error values and $${R}^{2}$$ for the permeability, calculated between the measured and predicted values. Table 7 presents some detailed statics for the predictive models generated (all data) using ANN and SVM, as well as statistics for the empirical models generated with LBM and well log data. These statistics show is closely related to the error analysis. According to the findings in Tables 6 and 7, the ANN predictive models outperformed the SVM models in terms of permeability prediction, because of their ability to adapt to complex and non-linear relationships within the data. This is important in carbonate rocks because they formed through a chemical diagenesis process which has more heterogeneity. The predictive ANN models exhibited smaller error values, with an MAE value of 2.166 for testing, and a standard error of 1.874 for testing. Additionally, we computed the error percentage for the predicted permeability values generated by both the ANN and SVM models. The results highlight that the ANN model exhibits a lower error percentage compared to the SVM model. The predicted permeability values generated by our ANN model have been effectively validated against well-log derived permeability values, specifically from well B in one of the study zones. Figure 24a provides a visual representation of this validation, depicting a cross plot between the ML based permeability and well log derived permeability. Remarkably, this comparison reveals highly promising results, with a coefficient of determination exceeding 0.86. This strong correlation underscores the reliability and accuracy of our ANN model’s permeability predictions. The analysis was performed through resampling of the data points of estimated permeability based on well data. Prior to this, the range of minimum and maximum values of well based estimated permeability was restricted in reference to the range of ML based estimated permeability. Figure 24b visually represents this error comparison between the ANN and SVM. However, it is important to note that the choice of model should be based on the specific situation and the required level of accuracy.

**Table 6:** Comparison of the ${R}^{2}$ values and error metrics of the SVM and ANN models to identify the better accuracy and reliability in predicting permeability.

Method	Dataset	${R}^{2}$	RMSE	MAE	MSE
SVM*	Training	0.859	2.281	1.842	5.204
	Testing	0.849	2.324	2.166	5.401
	Validation	0.860	2.226	1.567	4.955
	All data	0.849	2.229	1.736	4.972
ANN**	Training	0.955	1.272	0.98	1.619
	Testing	0.892	1.927	1.580	3.716
	Validation	0.908	1.650	1.405	2.722
	All data	0.921	1.533	1.228	2.352

*Support vector machine.

**Artificial neural network.

**Table 7:** Statistics on permeability results obtained by machine learning algorithms and empirical methods.

Dataset	Method	Arithmetic mean	Geometric mean	Median	Standard deviation	Standard error	Correlation coefficient
Well-A	Timur	15.56	1.10E−06	1.63E−06	125.968	4.521	
Well-B		10.04	0.016	0.0013	106.78	2.214	
Well-A & Well-B		11.67	0.00013	0.0006	113.11	2.053	
SEM	LBM	9.417	7.146	8.88	5.878	0.921	
Training	ANN	9.965	8.359	9.919	5.09	0.916	0.977
Training	SVM	9.315	7.639	8.51	7.175	0.998	0.926
Testing	ANN	10.112	7.972	6.967	3.456	1.447	0.944
Testing	SVM	8.924	7.630	8.16	4.591	1.874	0.921
Validation	ANN	6.138	4.278	6.742	4.340	1.772	0.953
Validation	SVM	8.108	7.603	11.68	5.238	2.138	0.927

## Conclusions

This study aimed to analyze 2D scanning electron microscope (SEM) images of carbonate samples, characterizing their pore structure. We developed a machine learning (ML) algorithm using pore network parameters as input data to predict the permeability. These predictions were compared with LBM simulation results, with the goal of establishing a reliable method for permeability prediction based on 2D SEM image analysis. The following conclusions are made based on the results of this study:

1. Initially, we investigated the impact of various threshold algorithms on gray scale images to effectively characterize the pore structure. We calculated porosity based on these algorithms, and MaxEntropy algorithm yielded results (0.02 to 0.12) that were closely aligned with petrographic studies (0.10 to 0.12).
2. We used a watershed algorithm at different magnifications to extract pore network parameters from the 2D SEM images of carbonate samples. At lower radii, we observed a higher number of pores and throats, whereas the number of pores and throats decreased as the radius increased. We also observed this pattern in the grain size distribution.
3. Magnification significantly influenced the pore network parameters. With increased magnification, the pore radius, throat radius, and grain size decreased. Conversely, the coordination number exhibited the opposite behavior, increasing with higher magnification.
4. We used Lattice Boltzmann Method (LBM) to estimate the permeability of carbonate samples. The Lattice Boltzmann Method (LBM) confirmed its validity and reliability in determining the permeability of carbonate samples by providing an acceptable value when compared with log-derived permeabilities.
5. Machine learning (ML) algorithms such as ANN and SVM methods offers a reliable and accurate approaches for permeability prediction models when using image extracted pore network parameters as input features and LBM permeability values as the output model. The evaluation of permeability prediction using the ANN method yielded notable results. The testing data’s coefficient of determination ($${R}^{2})$$ for the ANN approach was 0.892, with associated error values of 3.716 for MSE, 1.927 for RMSE and 1.580 for MAE. In contrast, when using the SVM method the $${R}^{2}$$ for testing data was 0.849, the corresponding error values were 5.401 for MSE, 2.324 for RMSE and 2.166 for MAE. These results indicate that the ANN approach outperforms the SVM method, demonstrating a higher level of accuracy in predicting permeability.

This research provides valuable insights with practical applications in the oil and gas industry, particularly in carbonate reservoirs. This study enhances our comprehension of these reservoirs by accurately predicting the permeability using ML algorithms. These insights can directly improve hydrocarbon exploration and production, optimize reservoir management, especially in carbonate reservoirs. The reliability of LBM simulations for permeability estimation reinforces its applicability, especially when dealing with heterogeneous carbonate samples.

## Data Availability

The data used in the present study was supported by Dr. Abhayanand Singh Maurya, Department of Earth Sciences at IIT Roorkee, India. It was collected from his published manuscript after proper permission. A suitable acknowledgment and citation of his published manuscripts have been mentioned in this study.
